# Fangchinoline Activates cGAS-STING to Promote Antitumor Immunity without Pathological Inflammation

**DOI:** 10.7150/ijbs.133255

**Published:** 2026-05-18

**Authors:** Yang Zhao, Xingyu Chen, Yunfei Xie, Tianyi Liu, Ying Luo, Zhengquan Liu, Yanyan Zhu, Lei Chen, Yuxiang Ren, Hanjie Liu, Pingsen Zhao, Qian Wang, Fuping You

**Affiliations:** 1 Institute of Systems Biomedicine, Department of Immunology, School of Basic Medical Sciences, Beijing Key Laboratory of Tumor Systems Biology, NHC Key Laboratory of Medical Immunology, Peking University Health Science Center, Beijing 100191, China.; 2 School of Pharmaceutical Sciences, Tsinghua University, Beijing 100084, China; Tsinghua-Peking Center for Life Sciences, Beijing 100084, China. Department of Laboratory Medicine, Yuebei People's Hospital Affiliated to Shantou University Medical College, Shaoguan 512025, China.; 3 State Key Laboratory of Natural and Biomimetic Drugs, School of Pharmaceutical Sciences, Peking University, Beijing, 100191, China.; 4 Yuquan Hospital, School of Clinical Medicine, Tsinghua Medicine, Tsinghua University, Beijing 100040, China.; 5 Department of Laboratory Medicine, Yuebei People's Hospital Affiliated to Shantou University Medical College, Shaoguan 512025, China.; 6 Department of Chemistry, Tsinghua University, Beijing 100084, China.

**Keywords:** fangchinoline, cancer immunotherapy, cGAS-STING, type I interferon, PD-1 blockade, hyperinflammation, epigenetic regulation, multi-omics

## Abstract

Effective cancer immunotherapy requires strengthening tumor-directed immunity while avoiding pathological inflammation, highlighting an urgent need for single agents that can achieve this balance. Here, we identified fangchinoline (Fan) as a dual immunomodulator that activated antitumor immune responses and restrained hyperinflammation. Fan induced robust type I interferon production across multiple human and murine cell types and in mice, and these responses were largely abolished by genetic deletion of cGAS or STING. Mechanistically, Fan directly bound and sensitized human cGAS and enhanced 2′,3′-cGAMP synthesis, including detectable activity in the absence of exogenous DNA, and this effect required an intact catalytic center. In vivo, Fan suppressed tumor growth in B16F10 melanoma and Pan02 pancreatic cancer models, increased intratumoral immune activation, and improved the efficacy of PD-1 blockade. Single-cell and multi-omics analyses further revealed coordinated transcriptional, chromatin-accessibility, and intercellular communication changes that supported enhanced CD8⁺ T cell effector programs within the tumor microenvironment. In parallel, Fan attenuated LPS-driven inflammatory responses in macrophages, reversed LPS-associated transcriptional and chromatin-opening programs, and improved survival in an endotoxemia model. Together, these findings established Fan as a cGAS-targeting immunomodulator that coupled antitumor immunity with control of inflammatory toxicity, providing a potential strategy to broaden the therapeutic window of immune activation.

## Introduction

Cancer immunotherapy has reshaped cancer treatment, yet durable responses remain limited to a subset of patients and tumor types 1-3. Effective tumor control often requires efficient antigen presentation and a T cell-inflamed tumor microenvironment 4, 5. Type I interferon is a key driver of these processes, as it supports dendritic cell activation, antigen cross-priming, and T cell recruitment into tumors 6-8.

The cyclic GMP-AMP synthase-stimulator of interferon genes pathway is a central axis that links cytosolic danger sensing to type I interferon induction 9-11. In cancer, this pathway can promote innate tumor sensing and amplify adaptive immunity 12-14. These features have motivated intensive efforts to develop pharmacologic activators of the cGAS-STING axis.

However, translating STING activation into safe and durable clinical benefit has been challenging 15. Systemic or excessive STING signaling can trigger broad inflammatory programs and cytokine elevation, which limits dosing and therapeutic index 16, 17. Even when delivered locally, STING agonists may still cause systemic immune activation in some settings, and clinical efficacy has often been modest 18-21.

A related challenge is the need to strengthen tumor-targeted immunity without provoking pathological inflammation outside the tumor. Several immune-stimulating approaches can enhance antitumor activity but also increase inflammatory toxicity. This tension is well recognized for innate immune agonists and for epigenetic strategies that induce viral-mimicry-like interferon programs 22-24.

Current approaches frequently rely on combining immunotherapies with anti-inflammatory agents, an approach that increases treatment complexity and can compound toxicity 25, 26. For example, combining anti-CTLA-4 with anti-PD-1 improves tumor control but causes high rates of immune-related adverse events, limiting its use to select patients 27-29. Conversely, mitigating immunotherapy-induced inflammation often requires systemic corticosteroids or cytokine antagonists, such as anti-TNFα, which may dampen antitumor immunity and cause additional adverse effects 30-32. In essence, approaches that can dissociate antitumor effects from inflammation are essential to improve the safety profile of immunotherapy 33-35. An ideal solution would be a single agent capable of simultaneously potentiating antitumor immunity and suppressing pathological inflammation, thereby avoiding the need for multi-drug regimens.

Here, we identified Fan as a small molecule activator of the cGAS-STING pathway with an unusual functional profile. We found that Fan induced robust type I interferon responses across multiple immune and non-immune cell types and triggered pathway activation *in vivo*. Using genetic loss of function models, we showed that this response depended on cGAS and STING. We then demonstrated direct binding of Fan to recombinant human cGAS and showed that Fan enhanced cGAS enzymatic activity *in vitro*, including measurable activation under conditions that normally leave cGAS inactive. Analysis of mutants further indicated that Fan-driven activation did not require the canonical DNA-binding interface but depended on an intact catalytic center.

We next explored whether this innate immune activation translated into antitumor benefit. In mouse tumor models, Fan suppressed tumor growth, increased immune infiltration, and enhanced antitumor effector programs. Fan also improved the therapeutic activity of PD-1 blockade. Moreover, we performed single cell transcriptomics on TILs and integrated these data with bulk RNA-seq and ATAC-seq of CD8+ TILs. These analyses revealed coordinated transcriptional and epigenetic remodeling consistent with enhanced immune activation in the tumor microenvironment. In parallel, Fan attenuated LPS-induced inflammatory genes and improved survival in an experimental sepsis setting.

Together, these findings established Fan as a direct cGAS-binding small molecule that boosted antitumor immunity while restraining excessive inflammation, and they supported further development of natural compounds as immunomodulators with improved therapeutic index.

## Results

### Fangchinoline Directly Binds and Sensitizes cGAS Activation to Induce Type I IFN Production

**Figure 1:**
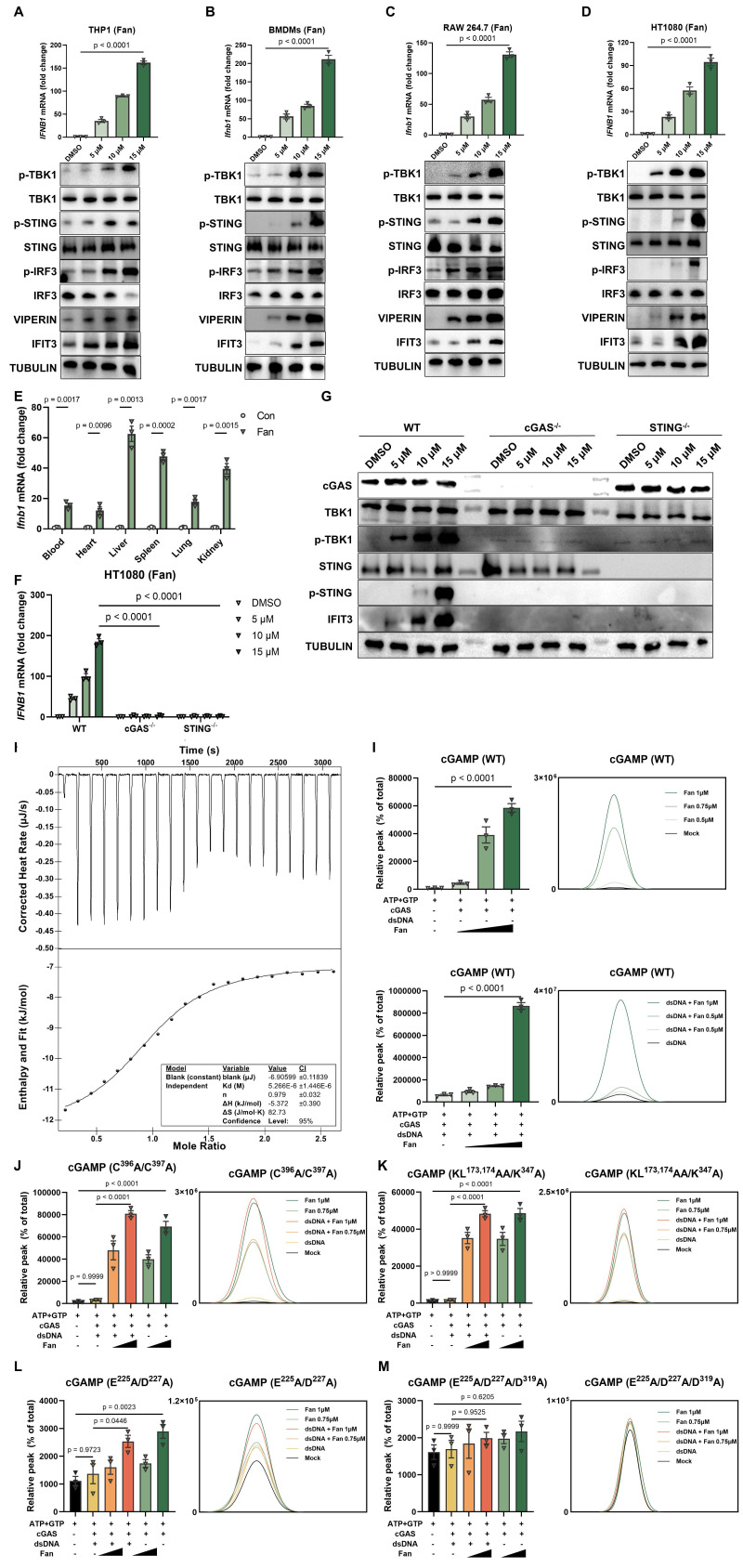
** Fangchinoline Directly Binds and Sensitizes cGAS Activation to Induce Type I IFN Production.** (A-D) Dose-dependent induction of *IFNB1* mRNA (RT-qPR, top panels) and corresponding protein responses (western blots, bottom panels) in THP1 (A), BMDMs (B), RAW264.7 (C) and HT1080 (D) cells treated with the indicated Fan concentrations for 12 hours. Ordinary one-way ANOVA test. (E) RT-qPCR quantification of *Ifnb1* mRNA in mouse bone marrow-derived macrophages (BMDMs) treated with indicated concentrations of Fan for 12 hours. Unpaired t test. (F) RT-qPCR of *IFNB1* mRNA in wild-type (WT), cGAS knockout and STING knockout HT1080 cells treated with Fan (5, 10 or 15 µM) or DMSO for 12 hours. Two-way ANOVA test. (G) Western blots of WT, cGAS knockout and STING knockout HT1080 cells treated as in (F). (H) Isothermal titration calorimetry (ITC) analysis showing the direct binding of Fan to recombinant human cGAS, with representative raw heat signals (top) and integrated binding isotherm fitted to a one-site binding model (bottom). (I) *In vitro* cGAS enzymatic assays: LC-MS quantification of cGAMP production by wild-type human cGAS (residues 157-522) incubated with dsDNA in the presence of Fan (0.5, 0.75 or 1 µM) or the absence of exogenous DNA. Ordinary one-way ANOVA test. (J-M) *In vitro* cGAS enzymatic assays: LC-MS quantification of cGAMP production by human cGAS mutants incubated with dsDNA in the presence of Fan (0.5, 0.75 or 1 µM) or the absence of exogenous DNA. The mutants included a Zn^2+^-thumb mutant (C396A/C397A) (J), a DNA-binding-interface mutant (K173A/L174A/K347A) with an N-terminal deletion (K), a catalytic-site double mutant (E225A/D227A) (L) and a catalytic-triad mutant (E225A/D227A/D319A) (M). Ordinary one-way ANOVA test. Data are shown as mean ± SEM.

During routine experiments, Fangchinoline (Fan) was repeatedly observed to robustly induce type I interferon responses. This effect was validated across multiple cell types, including THP-1, BMDMs, RAW 264.7, and HT1080 cells. RT-qPCR analysis showed a marked increase in *IFNB1* mRNA relative to the DMSO control. In parallel, western blot analysis revealed dose-dependent phosphorylation of TBK1 (p-TBK1), IRF3 (p-IRF3), and STING (p-STING), accompanied by increased expression of the interferon-stimulated genes Viperin and IFIT3 (Figure 1A-1D). Consistently, ELISA measurements demonstrated significantly elevated secretion of IFN-β in all Fan-treated cells (Figure S1A), supporting activation of the type I interferon program.

To further characterize the *in vitro* active concentration range of Fan, we performed additional dose-response and cell viability assays in representative cell types and determined the apparent EC50 and corresponding CC50 values (Figure S1D-S1E). These data supported that the concentrations used in this study were within an active range below overt cytotoxicity.

The interferon response induced by Fan was further examined *in vivo*. Six-week-old C57BL/6J mice received a single intraperitoneal injection of Fan (20 mg/kg). At 6 hours post-injection, RT-qPCR analysis showed significant systemic upregulation of *Ifnb1* mRNA in the heart, liver, lung, spleen, and kidney. In agreement, ELISA measurements detected increased IFN-β protein levels in both serum and tissues from Fan-treated mice (Figure 1E and Figure S1B). In addition, repeated administration of Fan for two weeks did not cause obvious body weight loss in mice, suggesting that Fan was generally tolerated under the tested conditions (Figure S1F).

To define the pathway responsible for Fan-induced innate immune activation, cGAS^-/-^ and STING^-/-^ HT1080 cell lines were generated using CRISPR/Cas9. Fan-induced *IFNB1* mRNA expression (Figure 1F) and secreted IFN-β (Figure S1C) were markedly reduced in both knockout cell lines compared with wild-type controls. Western blot analysis further showed that Fan-induced phosphorylation of STING and TBK1 was strongly attenuated in the absence of cGAS or STING (Figure 1G). These results indicated that Fan activated type I interferon signaling predominantly through the cGAS-STING pathway.

Mechanistically, we performed isothermal titration calorimetry (ITC) to assess whether Fan directly engaged cGAS. Fan bound recombinant human cGAS with a dissociation constant of 5.3 μM (Figure 1H). To evaluate whether Fan modulated cGAS catalytic activity, *in vitro* reconstitution assays were performed using purified human cGAS in the presence of ATP, GTP, and Mg²⁺, then 2′,3′-cGAMP production was quantified by LC-MS/MS. In the presence of dsDNA, co-incubation with Fan significantly enhanced 2′,3′-cGAMP production compared with dsDNA alone. Notably, in the absence of exogenously added DNA, induced a low but detectable level of 2′,3′-cGAMP, whereas cGAS remained inactive in control reactions (Figure 1I). These data supported a model in which Fan promoted cGAS activation and can partially bypass the requirement for an exogenous DNA trigger.

To delineate structural determinants required for Fan-mediated activation, a panel of cGAS mutants was evaluated. As expected, dsDNA failed to stimulate the Zn-thumb mutant (C396A/C397A) and the DNA-binding mutant (KL173,174AA/K347A). In contrast, Fan still induced a clear, dose-dependent increase in 2′,3′-cGAMP in both mutants (Figure 1J-1K), indicating that Fan-driven activation was not dependent on the canonical dsDNA-binding interface. For the catalytic double mutant E225A/D227A, Fan produced only a partial increase in 2′,3′-cGAMP (Figure 1L). Importantly, Fan-induced activity was abolished in the catalytic triple mutant E225A/D227A/D319A (Figure 1M). Collectively, these results demonstrated that Fan activated cGAS in a dsDNA-interface-independent manner while remaining strictly dependent on an intact catalytic center.

### Fangchinoline Promotes Antitumor Immune Responses to Enhance PD-1 Therapy

**Figure 2:**
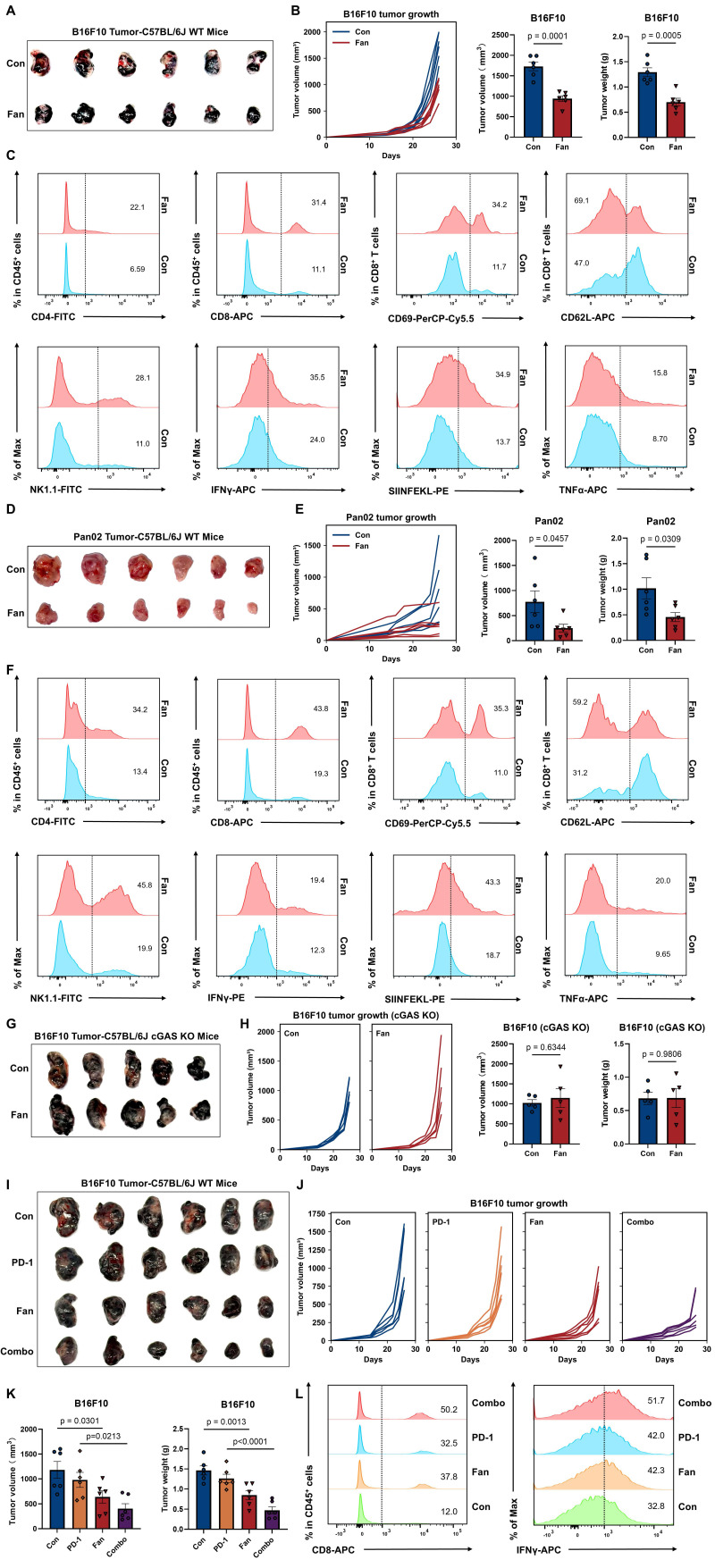
** Fangchinoline Promotes Antitumor Immune Responses to Enhance PD-1 Therapy.** (A-B) Wild-type (WT) mice were inoculated with the indicated numbers of B16F10 cells subcutaneously. Tumorigenesis was monitored every other day for 26 days. Representative images of tumors, tumor sizes and tumor weights in PBS-treated control (Con) and Fan-treated group. Unpaired t test. (C) Representative FACS data of tumor infiltrating CD45^+^ CD4^+^ TILs, CD45^+^ CD8^+^ TILs, CD69^+^ CD8^+^ TILs, CD62L^-^ CD8^+^ TILs, NK1.1^+^ TILs, IFNγ^+^ TILs, SIINFEKL^+^ TILs and TNFα^+^ TILs of mice as in A. (D-E) Wild-type (WT) mice were inoculated with the indicated numbers of Pan02 cells subcutaneously. Tumorigenesis was monitored every other day for 26 days. Representative images of tumors, tumor sizes and tumor weights in PBS-treated control (Con) and Fan-treated group. Unpaired t test. (F) Representative FACS data of tumor infiltrating CD45^+^ CD4^+^ TILs, CD45^+^ CD8^+^ TILs, CD69^+^ CD8^+^ TILs, CD62L^-^ CD8^+^ TILs, NK1.1^+^ TILs, IFNγ^+^ TILs, SIINFEKL^+^ TILs and TNFα^+^ TILs of mice as in D. (G-H) cGAS knockout mice were inoculated with the indicated numbers of B16F10 cells subcutaneously. Tumorigenesis was monitored every other day for 26 days. Representative images of tumors, tumor sizes and tumor weights in PBS-treated control (Con) and Fan-treated group. Unpaired t test. (I-K) Tumor sizes of subcutaneous B16F10 implanted in mice treated with the isotype antibody (200 μg/mouse i.p.), Fan (20 mg/kg i.p.), anti-PD-1 antibody (200 μg/mouse i.p.) or Fan plus anti-PD-1 antibody (Combo, combined treatment with Fan and anti-PD-1 antibody). Tumorigenesis was monitored every other day for 26 days. Representative images of tumors, tumor sizes and tumor weights. Ordinary one-way ANOVA test. (L) Representative FACS data of tumor infiltrating CD45^+^ CD8^+^ TILs and IFNγ^+^ TILs of mice as in I. Data are shown as mean ± SEM.

Based on our previous findings, we hypothesized that Fan might also enhance antitumor immunity. To test this, B16F10 cells were subcutaneously inoculated into wild-type C57BL/6J mice, followed by Fan treatment every other day (Figure S2A). Representative tumor images showed an obvious reduction in tumor size in the Fan-treated group compared with vehicle controls (Figure 2A). Consistently, tumor growth curves demonstrated that Fan significantly inhibited tumor progression, resulting in reduced tumor volume and lower tumor weight at endpoint (Figure 2B).

To characterize immune changes within the tumor microenvironment, immune cell populations in B16F10 tumors were analyzed by flow cytometry. Fan treatment increased the infiltration of CD4^+^ and CD8^+^ tumor-infiltrating leukocytes (TILs) compared with controls. Among these CD8^+^ TILs, BT markedly increased the proportion of cells expressing CD69, an early activation marker indicating recent T-cell receptor stimulation. Fan treatment also increased the percentage of CD62L⁻CD8⁺ TILs, a phenotype representing effector T cells that have lost lymph node-homing capacity and are associated with active antitumor responses. We also evaluated innate immune cell activation within the tumor microenvironment. Fan treatment significantly elevated the infiltration of NK1.1⁺ NK cells, which were innate lymphoid cells critical for initial tumor control. Functionally, Fan increased the SIINFEKL staining signal and enhanced IFN-γ and TNF-α production, further confirming increased antitumor immune activity (Figure 2C and S2B).

To test whether Fan exerted antitumor activity beyond the B16F10 melanoma model, we extended the analysis to the Pan02 pancreatic tumor model. Wild-type C57BL/6J mice were subcutaneously inoculated with Pan02 cells and treated with Fan on the same schedule as in the B16F10 experiments. Fan treatment markedly attenuated Pan02 tumor growth and significantly reduced endpoint tumor volume and tumor weight (Figure 2D-2E). Consistent with the B16F10 model, Fan increased immune infiltration in Pan02 tumors, with higher frequencies of CD4⁺ and CD8⁺ TILs as well as NK1.1⁺ NK cells. CD8⁺ TILs also displayed a more activated and effector-like state, showing increased CD69 expression and a higher proportion of CD62L-negative cells. Moreover, Fan-treated tumors produced more IFN-γ, SIINFEKL and TNF-α (Figure 2F and S2C).

Collectively, these results indicated that Fan promoted adaptive T cell responses and boosted innate immunity, thus shaping a more activated tumor immune microenvironment. Consistent with cGAS dependence, BT failed to attenuate tumor growth and increase infiltration of CD8⁺ TILs, IFN-γ or TNF-α production in cGAS-knockout mice (Figure 2G-2H and S2D).

To further explore the therapeutic potential of Fan in combination with immune checkpoint blockade therapy, we treated mice bearing B16F10 tumors with Fan, PD-1 antibody, or their combination. Combination therapy significantly inhibited tumor growth compared to either single treatment or control (Figure 2I-2J). Flow cytometry analysis of tumors showed a marked increase in total CD8⁺ TILs, IFN-γ⁺ and TNF-α populations (Figure 2K-2L and S2E-S2F), demonstrating enhanced cytotoxic function induced by combined treatment.

To extend our *in vivo* evaluation beyond subcutaneous tumor models, we further examined the effect of Fan in a B16F10 lung metastasis model. Fan treatment significantly reduced pulmonary metastatic burden, supporting its antitumor activity in a metastatic setting (Figure S2G).

### Fangchinoline Enhances CD8⁺ TIL Effector Programs via Transcriptional and Epigenetic Remodeling

To comprehensively understand how Fan potentiated antitumor immunity, we performed single-cell RNA sequencing (scRNA-seq) on CD45⁺ tumor-infiltrating leukocytes (TILs) from Fan-treated and PBS-treated (Con) B16F10 tumors. After standard preprocessing and quality control (QC), violin plots of key QC metrics showed overall comparable data quality between the two groups (Figure S3A).

**Figure 3:**
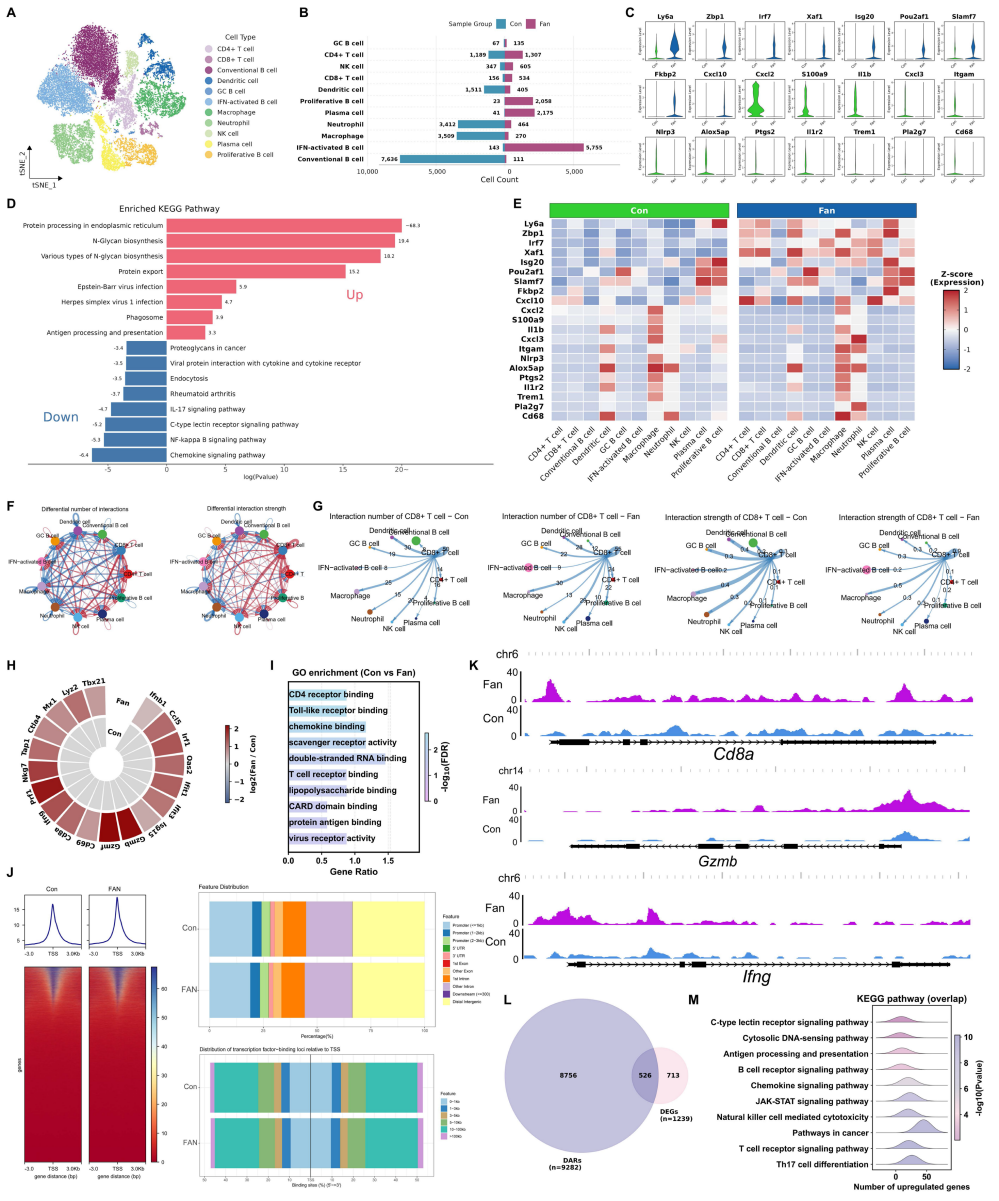
**Fangchinoline Enhances CD8⁺ TIL Effector Programs via Transcriptional and Epigenetic Remodeling.** (A) t-SNE plot illustrating cell population clustering and annotation in CD45^+^ B16F10 tumor samples treated with PBS (Con) or Fan, with clusters color-coded by cell type. (B) Bar chart comparing the proportions of major cell types between Fan- and PBS-treated groups in CD45^+^ B16F10 tumors. (C) Violin plots comparing the expression of critical genes in Fan- and PBS-treated CD45^+^ B16F10 tumor cells. (D) KEGG pathway analysis of differentially expressed genes (DEGs) in CD45^+^ cells following Fan treatment compared to Con. (E) a heatmap of selected immune-related genes across different cell populations under control (Con) and Fangchinoline (Fan) treatment conditions. (F) Interaction network between different cell types in the tumor microenvironment, showing predicted interactions. Line thickness indicated the number of interactions or the strength of interactions. (G) Cell-cell communication analysis centered on CD8+ T cells under Con and Fan treatment conditions. (H-I) Heatmap of DEGs in CD8^+^ TILs of B16F10 tumors and Gene Ontology (GO) pathway analysis of DEGs. (J) ATAC-seq metaprofile and heatmap of chromatin accessibility ± 3 kb around peak centers in PBS- and Fan-treated groups (left). Distance-to-TSS annotation and genomic feature distribution of ATAC peaks in Fan and PBS samples (right). (K) Visualization of peak tracks for the PBS and Fan groups using IGV software. (L) Venn diagram showed overlap of upregulated genes between ATAC-seq and bulk RNA-seq. (M) KEGG enrichment analysis of overlap genes in L.

We visualized the integrated CD45+ TIL transcriptomes using both t-distributed stochastic neighbor embedding (t-SNE) and uniform manifold approximation and projection (UMAP), with cells colored by treatment group, to assess the overall distribution and mixing of Con and Fan cells (Figure S3B and S3L). We then performed sub-clustering and cell-type annotation to evaluate Fan-associated gene-expression and pathway changes within each immune subpopulation (Figure S3C). Unsupervised clustering using t-SNE of TILs analysis identified eleven major immune cell populations, including CD4+ T cells, CD8+ T cells, conventional B cells, dendritic cells (DCs), GC B cells, IFN-activated B cells, macrophages, neutrophils, NK cells, plasma cells and proliferative B cells (Figure 3A). In Fan-treated tumors, we observed significant compositional changes in immune cell populations compared to controls (Figure S3D). Fan decreased conventional B cells but increased IFN-activated B cells, indicating a shift in B-lineage composition. Fan also increased dendritic cells and NK cells while reducing tumor-associated neutrophils (TANs) and macrophages (Figure 3B and S3E). Compared with controls, Fan treatment increased the expression of interferon-stimulated genes such as Zbp1, Irf7, Xaf1, and Isg20, and also elevated inflammatory and myeloid activation programs marked by Cxcl10, Il1b, S100a9, Nlrp3, and Ptgs2, indicating strengthened IFN-linked inflammatory signaling in tumor-infiltrating immune cells (Figure 3C and S3F). Gene Ontology (GO) and Kyoto Encyclopedia of Genes and Genomes (KEGG) pathway enrichment analysis of differentially expressed genes (DEGs) revealed an upregulation of immune activation-related pathways (Figure 3D and S3G). Moreover, multiple interferon response genes showed higher standardized expression in Fan versus control samples across several major cell types (Figure 3E). This pattern indicated that Fan enhanced an IFN-associated inflammatory gene program in a cell-type-resolved manner. CellChat analysis revealed enhanced intercellular communication within the tumor immune microenvironment following Fan treatment. The interaction network diagrams clearly illustrated the increased numbers and strengths of cellular interactions compared to controls, predominantly driven by CD8+ T cells (Figure 3F-3G and S3H-S3J). Together, these results suggested that Fan enhanced immune cell crosstalk and activated multiple communication pathways linked to antitumor immunity.

To further validate these findings at the cell population level, we sorted CD8⁺ TILs from B16F10 tumors and performed bulk RNA-seq to confirm their transcriptional activation. Fan-treated CD8+ T cells displayed higher expression of interferon-response genes than controls, including Ifnb1, Irf1, Oas2, Ifit1, Ifit3, Isg15 and Mx1. Genes linked to cytotoxic effector programs were also increased, including Prf1, Nkg7, Gzmb and Ifng, together with elevated Ccl5 and Tap1. In addition, Cd69 and Tbx21 were increased, and Ctla4 was also higher, indicating concurrent induction of activation- and checkpoint-associated transcriptional programs (Figure 3H). GO enrichment analysis of CD8+ TILs sorted from B16F10 tumors highlighted Toll-like receptor binding, T cell receptor binding and virus receptor activity (Figure 3I). KEGG enrichment highlighted cytosolic DNA-sensing pathway and PD-L1 expression and PD-1 checkpoint pathway in cancer (Figure S3K).

We next examined whether the transcriptional enhancements in Fan-treated T cells were accompanied by epigenetic remodeling. Assay for transposase-accessible chromatin using sequencing (ATAC-seq) on CD8+ TILs demonstrated that Fan treatment broadly increased chromatin accessibility across the genome, with a marked enrichment of open chromatin at gene promoter regions (Figure 3J). Genome browser views (IGV) illustrated increased the accessibility of key interferon stimulated genes at representative loci, such as Cd8a, Gzmb and Ifng, consistent with their transcriptions (Figure 3K). Integrative analysis intersecting the ATAC-seq data with the bulk RNA-seq results revealed that 526 upregulated in Fan-treated CD8+ T cells also displayed increased promoter accessibility (Figure 3L). KEGG enrichment of these overlapping genes with concomitant chromatin opening showed significant enrichment in immune-related pathways, including the cytosolic DNA-sensing pathway, JAK-STAT signaling pathway, and T cell receptor signaling pathway (Figure 3M).

Collectively, these multi-omic results demonstrated that Fan therapy led to a multi-level enhancement of anti-tumor immunity in the tumor microenvironment. Fan treatment not only reshaped the cellular composition of TILs and boosted T cell functional gene expression, but also established an epigenetic landscape conducive to robust immune activation. In summary, Fan exerted broad immune regulatory effects, promoting a more immunologically active tumor microenvironment through coordinated changes at the cellular, molecular, and chromatin levels.

### Fangchinoline Suppresses LPS-Induced Inflammation

**Figure 4:**
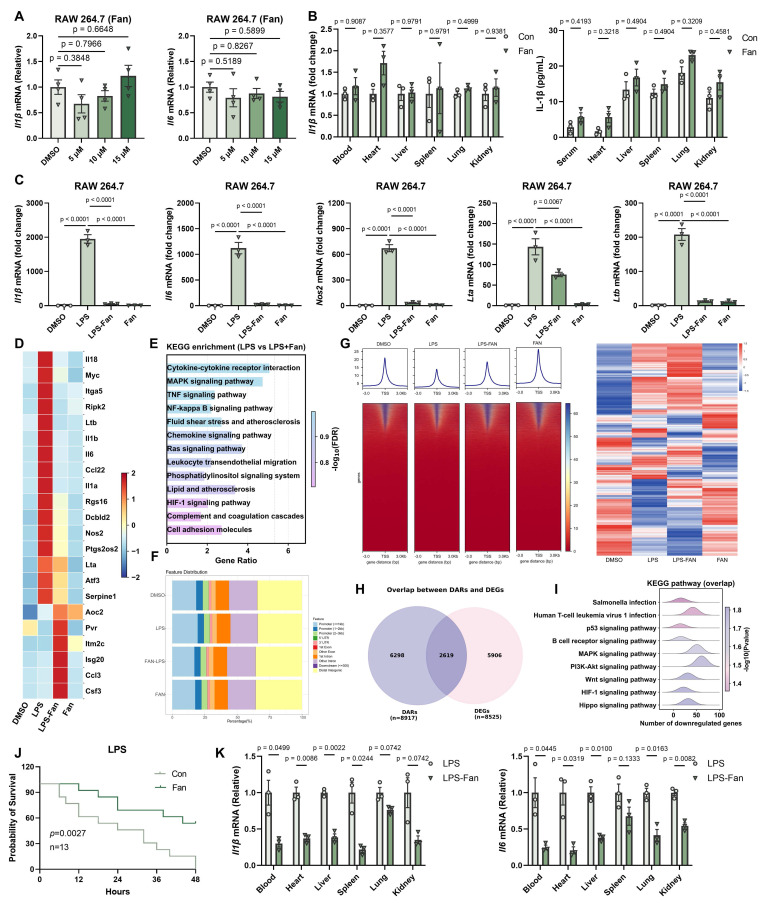
** Fangchinoline Suppresses LPS-Induced Inflammation.** (A) RT-qPCR of *Il1b* and *Il6* in RAW 264.7 cells following Fan treatment (5, 10, 15 µM) for 12 hours. Ordinary one-way ANOVA test. (B) *In vivo* detection of *Il1b* mRNA and Elisa of IL-1β in blood, heart, liver, lung, spleen and kidney of C57BL/6J mice 6 hours after a single intraperitoneal injection of Fan (20 mg/kg). Unpaired t test. (C) Fan markedly downregulated the expression of *Il1β*, *Il-6*, *Nos2*, *Lta*, *Ltb* mRNA in RAW264.7 cells, following treatment with DMSO, LPS, LPS-Fan and Fan alone. Ordinary one-way ANOVA test. (D) Heatmap illustrated the expression of regulated genes in the Fan group compared to DMSO groups, specifically those involved in the inflammatory response pathway. (E) KEGG enrichment of differentially expressed genes (DEGs) between the treatment of LPS with Fan or LPS alone in RAW264.7 cells. (F) Distribution of peaks and transcription factor-binding loci relative to transcription start sites (TSS) in the LPS, LPS-Fan, and Fan groups compared to the DMSO group, respectively, as annotated using the ChIPseeker R package. (G) Heatmap showed that Fan markedly changed the chromatin accessibility induced by LPS (left). Heatmap illustrated the differentially accessible regions (DARs) most prominent in the LPS group compared to other groups (right). (H) Venn diagram showed overlap of upregulated genes between ATAC-seq and bulk RNA-seq. (I) KEGG enrichment analysis of overlap genes in H. (J) Fan significantly decreased the mortality rate in mice (n=13) following LPS challenge. Log-rank (Mantel-Cox) test. (K) RT-qPCR quantification of *Il1β* and *Il-6* in blood, heart, spleen, lung, kidney, liver. Unpaired t test. Data are shown as mean ± SEM.

During our daily experiment, we found Fan cannot induce inflammatory *in vitro* and *in vivo* (Figure 4A-4B). Given the broad immunomodulatory effects of Fan observed in tumor models, we next determined whether Fan could also suppress excessive inflammatory responses. We first examined its impact using an *in vitro* LPS-induced inflammation model. RAW264.7 macrophages were treated with DMSO, LPS, LPS+Fan, or Fan alone. RT-qPCR analysis showed that LPS stimulation robustly upregulated the *Il1b*, *Il6*, *Nos2*, *Lta* and *Ltb*, compared to DMSO controls (Figure 4C). Co-treatment with Fan significantly decreased the LPS-induced transcription of these genes. Fan alone had little effect on the basal expression of these inflammatory markers. Importantly, ELISA analysis of culture supernatants further demonstrated that Fan reduced LPS-induced IL-1β protein secretion (Supplementary Figure 4A). Thus, Fan markedly attenuated LPS-triggered inflammatory gene induction at the mRNA level in macrophages.

To comprehensively investigate Fan's effects on LPS-driven gene expression in RAW264.7 cells, we performed bulk RNA-sequencing on each sample. LPS alone triggered a broad inflammatory transcriptional program, with hundreds of genes differentially expressed relative to control. Fan co-treatment substantially altered this program. Heatmap visualization of differentially expressed genes (DEGs) indicated that the expression profile of Fan co-treated cells was distinct from LPS alone, characterized by lower expression of inflammatory genes (Figure 4D). KEGG enrichment highlighted multiple inflammation-related pathways among DEGs, including TNF signaling pathway and NF-kappaB signaling pathway (Figure 4E).

To explore the epigenetic basis of Fan's anti-inflammatory effects, we performed ATAC-seq to evaluate chromatin accessibility in RAW264.7 macrophages under the treatment of DMSO, LPS, Fan+LPS or Fan alone. LPS stimulation alone caused widespread opening of chromatin at regulatory regions, reflecting the activation of inflammatory gene loci. Remarkably, Fan co-treatment reversed a large portion of these changes, leading to reduced chromatin accessibility at multiple LPS-responsive loci (Figure 4F-4G).

Furthermore, we integrated the transcriptomic and epigenomic datasets to directly link Fan-induced changes in gene expression to alterations in chromatin state. We identified 2619 genes significantly downregulated at the mRNA level that concurrently showed reduced promoter accessibility based on ATAC-seq analysis (Figure 4H). KEGG pathway analysis of these overlapping genes revealed strong enrichment for inflammatory pathways, including p53 and MAPK signaling pathway, that were concordantly suppressed by Fan at both the chromatin and transcriptional levels (Figure 4I). This integrated multi-omics evidence firmly reinforced the conclusion that Fan suppressed LPS-induced inflammation in RAW264.7 cells.

Encouraged by the *in vitro* findings, we next evaluated whether Fan could ameliorate systemic inflammation *in vivo* using an LPS-induced sepsis model. Six-week-old C57BL/6J mice received the pretreatment of a single dose of Fan (20 mg/kg, i.p.) followed by a lethal intraperitoneal injection of LPS (20 mg/kg) one hour later. All vehicle-treated mice died within 48 hours, whereas nearly half of the Fan-treated mice survived (Figure 4J). To further assess systemic inflammation, we harvested blood, heart, spleen, lung, liver and kidney tissues 12 hours post-LPS injection and measured inflammatory cytokine mRNA levels. Fan-treated mice exhibited significantly decreased levels of *Il1β*, *Il6* mRNAs across all tested tissues compared to controls, demonstrating effective suppression of systemic inflammation (Figure 4K). Consistently, ELISA analysis further showed that Fan reduced IL-1β protein levels in the serum and in multiple organs of LPS-challenged mice (Supplementary Figure 4B). These results indicated that Fan effectively protected mice from hyperinflammation and endotoxic lethality, aligning with its potent anti-inflammatory action observed *in vitro*.

Together, our results demonstrated that Fan possessed potent anti-inflammatory activity, broadly suppressing LPS-induced inflammation through coordinated transcriptional repression and epigenetic modulation of critical inflammatory pathways. Given its ability to activate the cGAS-STING axis while restraining excessive inflammation, Fan may serve as a promising immunomodulatory candidate for future development as an antitumor immunotherapy adjuvant and as a therapeutic strategy for infection- or endotoxin-driven hyperinflammatory diseases.

## Discussion

In this study, Fangchinoline (Fan) acted as a dual immunomodulator that strengthened antitumor immunity while limiting excessive inflammation. Fan treatment inhibited tumor growth in mice models and increased immune activation in the tumor microenvironment. In parallel, Fan attenuated LPS-driven inflammatory programs in macrophages and improved survival in an endotoxic shock model. Together, these findings addressed a central barrier in immunotherapy, how to amplify protective immunity against tumors without escalating harmful systemic inflammation 36.

A key implication of our work was that Fan enhanced type I interferon signaling through the cGAS-STING axis. Type I interferon is a well-established driver of antitumor immunity because it supports dendritic cell maturation, antigen presentation, and priming of cytotoxic T cells. Many STING agonist strategies have leveraged this biology but have faced dose-limiting inflammation and systemic adverse events in early clinical studies, even when delivered intratumorally 20, 37-39.

Our results positioned Fan as a distinct small-molecule approach that robustly induced interferon programs while remaining compatible with an anti-inflammatory outcome under LPS-treatment. Mechanistically, Fan directly engaged cGAS and increased its catalytic output. The ITC measurements supported direct binding of Fan to recombinant human cGAS. Biochemical reconstitution further showed that Fan enhanced 2′,3′-cGAMP production in the presence of dsDNA and enabled detectable cGAMP generation even without exogenous DNA. This behavior contrasted with canonical cGAS activation, which typically requires dsDNA binding to trigger catalysis 40. The ability of a non-nucleotide small molecule to stimulate cGAS in a DNA-independent manner is notable, given that only a limited set of conditions have been shown to bypass the DNA requirement, such as manganese-enhanced cGAS activation 41-43. The mutant analysis further supported a model in which Fan did not rely on the canonical DNA interface but required an intact catalytic center, consistent with a direct catalytic sensitization mechanism.

Our work also extended prior observations on Fan and innate immunity. Recent studies reported that Fan enhanced antiviral responses in a STING-dependent manner and suggested that it could modulate STING stability. By defining direct cGAS binding and a DNA-interface-independent activation pattern, our data provided a complementary and more proximal mechanism that can explain robust induction of interferon-stimulated genes across multiple cell types and *in vivo*.

At the tumor level, single-cell and bulk profiling indicated that Fan remodeled immune states toward interferon related activation and enhanced immune crosstalk. Fan shifted B-lineage composition toward an interferon-activated state and expanded antigen-presenting and cytotoxic compartments, accompanied by increased interferon-stimulated gene expression across subsets. These changes aligned with the known role of interferon programs in shaping inflamed tumor microenvironments and improving responses to checkpoint blockade. In line with this concept, Fan improved the efficacy of PD-1 blockade in our B16F10 model.

An important and clinically relevant aspect of Fan was its anti-inflammatory activity in the LPS challenge setting. Fan suppressed LPS-induced cytokine gene induction, reversed inflammation-associated chromatin opening, and reduced systemic inflammatory burden *in vivo*. This phenotype is conceptually related to emerging efforts that use epigenetic control to tune immune outputs, including “viral mimicry” approaches that elevate interferon programs through endogenous RNA species 44-46.

Our data suggested that Fan could engage immune activation in tumors while restraining hyperinflammation in endotoxemia, supporting the feasibility of dissociating protective immunity from pathological inflammation.

Several limitations and future directions should be noted. First, although Fan bound human cGAS *in vitro*, the broader translatability to human primary immune and tumor samples needs to be established, including dose-response relationships and safety windows. Second, the structural basis of Fan-cGAS engagement remains to be defined. High-resolution structural studies and systematic binding-site mapping would clarify how Fan bypassed the DNA interface and could guide the design of derivatives with improved potency and selectivity. Third, although Fan directly bound cGAS and activated cGAS-STING signaling in the context of type I interferon induction, the current study did not establish whether its anti-inflammatory effect during LPS stimulation was mediated by the same pathway. Whether the LPS-suppressive effect of Fan requires cGAS-STING, or instead involves additional targets in macrophages, warrants further investigation. Fourth, although Fan was generally tolerated under the dosing conditions used in this study, a more detailed investigation of its toxicological properties will be an important focus of our future work and will be addressed more systematically in a subsequent study. In conclusion, our study identified Fan as a small molecule that directly bound cGAS, increased cGAMP production, activated type I interferon programs, and enhanced antitumor immunity while suppressing LPS-driven hyperinflammation. These findings established Fan as a mechanistically defined immunomodulator and suggested a broader strategy for developing cGAS-targeting agents that promote antitumor efficacy while limiting inflammatory toxicity, a balance that remains difficult to achieve with current innate immune agonists.

## Material and Methods

### Reagents and antibodies

Rabbit antibodies against TBK1 (catalog no. 28397-1-AP), Beta Tubulin (catalog no. 10094-1-AP) and IFIT3 (catalog no. 15201-1-AP), along with mouse antibodies targeting Beta Actin (catalog no. 66009-1-Ig), were procured from Proteintech. Additional antibodies, including rabbit phospho-TBK1/NAK (Ser172) (D52C2) (catalog no. 5483), phospho-IRF-3 (Ser396) (D6O1M) (catalog no. 29047), IRF-3 (D6I4C) (catalog no. 11904), cGAS (D1D3G) (catalog no. 15102), STING (D2P2F) (catalog no. 13647) and Viperin (F7T8D) (catalog no. 75654) were obtained from Cell Signaling Technology. Secondary antibody utilized HRP-conjugated Affinipure Goat Anti-Rabbit IgG (H+L) (catalog no. SA00001-2) and HRP-conjugated Affinipure Goat Anti-Mouse IgG (H+L) (catalog no. SA00001-1), were also from Proteintech. All fluorochrome-conjugated flow cytometry antibodies were bought from Biolgend. ABclonal supplied active recombinant mouse granulocyte-macrophage colony-stimulating factor (GM-CSF) protein (catalog no. RP01206). Fangchinoline (catalog no. HY-N1372A) and lipopolysaccharides, from E. coli O55:B5 (catalog no. HY-D1056) was bought from MedChemExpress.

### Cells

The RAW264.7 (RRID: CVCL_0493), THP-1 (RRID: CVCL_0006), HT1080 (RRID: CVCL_0316) was sourced from the American Type Culture Collection (ATCC), and maintained in Dulbecco's Modified Eagle's Medium (DMEM) (03.1002C, EallBio). Bone marrow-derived macrophages (BMDMs) were isolated from the femurs and tibiae of 8-week-old C57BL/6J mice. The bone marrow was cultured with GM-CSF for 7 days to induce macrophage differentiation. Culture media were supplemented with 10% fetal bovine serum (FBS) and 1% penicillin-streptomycin for optimal cell growth and maintenance. Additionally, cell identity was verified by short tandem repeat (STR) profiling, and routine mycoplasma screening with Mycolor One-Step Mycoplasma Detector (Vazyme, D201-01) was consistently negative.

### Mice

Wild-type (WT) C57BL/6J mice were purchased from the Department of Laboratory Animal Science, Peking University Health Science Center. cGAS knockout mice, all on a C57BL/6J background, were gifts from Zhengfan Jiang (Peking University).

All animal care and procedures were conducted in accordance with the Guide for the Care and Use of Laboratory Animals by the Chinese Association for Laboratory Animal Science. The protocols were approved by the Animal Care Committee of Peking University Health Science Center (permit number: LA 2016240). Mice were bred and housed under specific pathogen-free conditions at the Laboratory Animal Center of Peking University. Only mice aged 6 to 8 weeks were used in the experiments.

### Construction of a sepsis model via LPS injection

Sepsis induced by lipopolysaccharide (LPS) was used to model systemic inflammatory responses. Male C57BL/6J mice aged 6 to 8 weeks and weighing 20 to 25 grams were randomly assigned to two groups with twelve animals per group, an LPS plus PBS group and an LPS plus Fan group. LPS at 20 mg/kg in sterile saline was delivered by intraperitoneal injection using sterile insulin syringes. Immediately after LPS administration, mice in the Fan group received an intraperitoneal dose of 100μL Fan (20 mg/kg), while control mice received an equal volume of PBS. Survival was monitored for 48 hours, and blood and tissues were collected 12 hours after LPS administration for downstream analyses.

### Tumor transplantation and treatments

Mice were subcutaneously injected into the right flank with 5 × 10⁵ Pan02 cells or B16F10 cells suspended in 100 μL PBS, unless otherwise specified. Tumor growth was monitored daily, with tumor size measured every 2-3 days. Tumor volume was calculated using the formula: volume = length (mm) × width² (mm²) × 0.5. Tumor-bearing mice were treated with 20 mg/kg Fan or the same volume PBS via intraperitoneal injection every 2 days. For immune checkpoint blockade, tumor-bearing mice were administered 200 μg of anti-mouse PD-1 antibody (200 μL saline) via intraperitoneal injection on days 3, 7, 11 and 15 following tumor inoculation. Control mice received 200 μL of rat IgG2a isotype (Clone 2A3, BioXCell) on the same schedule.

### Tissue digestion and isolation of tumor-infiltrating leukocytes

Tumors were collected into cold PBS containing 1% heat-inactivated serum (FACS), then minced and digested in RPMI 1640 medium containing 0.5 mg/mL collagenase D (11088866001, Roche) and 0.1 mg/mL DNase I (DN25-100G, Sigma-Aldrich) at 37°C for 1 hour.

Digests were filtered through a 100 μm strainer into cold FACS buffer, further passed through a 200 μm mesh, and centrifuged at 1600 rpm for 5 minutes. Pellets were gently resuspended in Ficoll according to the manufacturer's instructions and overlaid with serum-free medium. After centrifugation at 800 × g for 20 minutes with low acceleration and deceleration, the mononuclear cell layer at the interface was collected, washed with FACS buffer, and centrifuged at 1600 rpm for 5 minutes. Cells were resuspended in 80% Percoll. Where indicated, leukocytes were enriched on a discontinuous Percoll gradient by gently layering 40% Percoll above the cell suspension and serum-free medium on top. The gradient was centrifuged at 800 × g for 30 minutes at room temperature with low acceleration and deceleration. Cells at the interface between 80% and 40% Percoll were collected, washed with FACS buffer. Red blood cells were lysed in ACK lysis buffer for 2 minutes, followed by neutralization with FACS buffer and centrifugation. The resulting cell mixture was then filtered through a 70-μm cell strainer to obtain a single-cell suspension, and then counted cells. Only samples with cell viability above 90% proceeded to flow cytometry.

### Flow cytometry analysis

Single-cell suspensions were kept on ice. Cells were stained with fluorochrome-conjugated antibodies against surface markers in FACS buffer for 20 to 30 minutes in the dark, then washed and resuspended. For intracellular cytokines, cells were stimulated with PMA and ionomycin in the presence of brefeldin A where indicated, fixed and permeabilized using standard buffers, and stained for IFN-γ and TNF-α. Data were acquired on a calibrated flow cytometer (BD Biosciences) using matched compensation controls, fluorescence-minus-one controls, and live-dead discrimination. Analyses were performed in FlowJo.

### RNA extraction, reverse transcription, and quantitative PCR

Total RNA was extracted from cells after the indicated treatments or infections using TRIzol reagent from TIANGEN, catalog A0123A01. RNA was reverse transcribed to cDNA with HiScript II RT SuperMix from Vazyme, catalog R223-01. Target transcripts were quantified by real-time PCR using SYBR Green qMix from Vazyme, catalog Q311. Relative mRNA levels were normalized to Actb as the housekeeping gene. Primer sequences are listed in Supplementary Table S1.

### Total protein extraction and immunoblotting

Cells were lysed in RIPA lysis buffer, strong formulation, from MedChemExpress, catalog HY-K1001. The buffer contained an EDTA-free protease inhibitor cocktail and phosphatase inhibitor cocktails II and III at 1:100 in DMSO. Lysates were cleared by centrifugation, and protein concentration was measured. Equal amounts of protein, typically 10 to 30 micrograms, were separated by SDS-PAGE and transferred to nitrocellulose membranes from Beyotime, catalog FFN08. Membranes were blocked, incubated with primary antibodies, washed, and then incubated with HRP-conjugated secondary antibodies. Signals were developed by enhanced chemiluminescence using the ECL kit from EallBio, catalog 07.10009-50, and recorded on a digital imager.

### Expression and purification of cGAS truncations and mutations

cGAS truncations and mutations were PCR-amplified from plasmid pET28a+-cGAS, which carried the full-length human cGAS gene or its mutants, and cloned into the pET-28a+ vector optimized for E. coli expression of an N-terminal 6xHis-SUMO-TEV fusion protein. Recombinant proteins were expressed in ER2566 (WEIDI) at 16 °C after initial growth in LB medium at 37 °C for 8 h and subsequent induction with 0.5 mM IPTG at 16 °C for 14 h. Cells were harvested by centrifugation at 1,914 × g for 10 min at 4 °C, resuspended in 25 mM Tris-HCl (pH 7.5), 20 mM imidazole, 500 mM NaCl, 3 mM β-mercaptoethanol and 2 mM PMSF, and lysed by high-pressure homogenization (Union-Biotech) at 800 bar for 10 min at 4 °C. The lysate was clarified by centrifugation at 34,571 × g for 30 min, and the supernatant was applied to a Ni-NTA column (Qiagen) for 1 h at 4 °C. Bound protein was eluted with lysis buffer supplemented to 100 mM imidazole, the SUMO tag was removed by TEV protease digestion, and the best fractions were pooled, concentrated and further purified by size-exclusion chromatography on a Superdex 200 10/300 GL column (Cytiva) in 50 mM Tris-HCl (pH 8.0), 300 mM NaCl.

### cGAS activity assay

Purified human cGAS (hcGAS) was incubated at 37 °C for 30 minutes in a 50 μL reaction that contained 1 μM hcGAS, 1 mM ATP, 1 mM GTP, 100 mM NaCl, 40 mM Tris-HCl at pH 7.5, and 10 mM MgCl₂. Where indicated, the reaction included double-stranded DNA at 5 μg/mL. Reactions were heated to 99 °C, then 200 μL acetonitrile was added to precipitate proteins. Samples were centrifuged at 14,000 rpm for 40 minutes, and the supernatants were analyzed by LC-MS/MS.

### LC-MS/MS quantification of 2′,3′-cGAMP

Mass spectrometry was performed on a Xevo TQ-S triple-quadrupole instrument with electrospray ionization in positive mode. Samples were separated on a reverse-phase C18 column with a mobile-phase flow rate of 0.3 mL/min. Mobile phase A was water with 0.1% formic acid, and mobile phase B was acetonitrile with 0.1% formic acid. The gradient started at 2% B for 1 minute, increased linearly to 98% B over 4 minutes, was held for 1 minute, and then returned to 2% B over 0.5 minute. Data were acquired in multiple-reaction-monitoring mode. The precursor ion at m/z 675.1 was monitored with two product ions: 675.1→136.0 for quantification and 675.1→152.0 for confirmation.

### RNA-seq and data analysis

Total RNA was extracted with the TIANGEN A0123A01 high-throughput kit. Quality control, library preparation, sequencing and downstream analysis were performed by Suzhou GENEWIZ following their standard protocols. Gene counts were generated by featureCounts v2.0.0 and normalized to FPKM. Differential expression was assessed in DESeq2 v1.38.3 using |log₂FC|>2 and p<0.05.

### ATAC-seq and data analysis

We performed ATAC-seq using the Hyperactive ATAC-Seq Library Prep Kit (TD711, Vazyme Biotech). Tn5 transposase, preloaded with sequencing adapters, was incubated with isolated nuclei to insert adapters into open chromatin. After PCR amplification with indexed primers, libraries were sequenced by GENEWIZ (Suzhou, China), yielding a genome-wide map of chromatin accessibility.

Raw ATAC-seq reads (paired-end) from GENEWIZ were first trimmed with Trim-Galore v0.6.4, then aligned to the mouse mm10 genome using Bowtie2 v2.3.5.1 (--very-sensitive -X 2000) and piped through SAMtools v1.10 to produce sorted, indexed BAM files. Peaks were called for each sample with MACS3 v3.0.0a5 (default settings), yielding per-sample BED files.

For ATAC-seq, individual peak sets were merged across all samples with Bedtools v2.31.1, and genome-wide coverage tracks were generated in RPKM units using deepTools v3.3.2 (bamCoverage --binSize 100 --normalizeUsing RPKM --effectiveGenomeSize 2864785220 --ignoreForNormalization chrM --extendReads). Differential accessibility/enrichment analyses were performed in R using the csaw package v1.38.0, and peaks were annotated with ChIPseeker v1.34.1. Final track visualization was carried out in IGV v2.17.4.

### Generation and processing of single-cell RNA sequencing data

Tumors were harvested after subcutaneous implantation and were gently dissociated to single-cell suspensions. The cell suspensions were transferred to Genewiz, Azenta Life Sciences. CD45 positive cells were isolated by flow cytometric sorting and were used for library preparation. Libraries were prepared with the 10x Genomics Chromium Next GEM Single Cell 3′ Reagent Kits v3.1 according to the manufacturer's instructions. Sequencing was carried out on an Illumina NovaSeq 6000 in paired-end 150 base-pair mode. Genewiz delivered FASTQ files for analysis.

Raw reads were processed with Cell Ranger version 3.1.0 for barcode demultiplexing, alignment, and unique molecular identifier counting to generate feature-barcode matrices. Downstream analyses were performed in R with Seurat version 4.3.0. Data were normalized, subjected to dimensionality reduction, and clustered to define cell populations. Cell types were annotated manually based on canonical marker genes. Results were exported for subsequent analyses.

Cell-cell communication was assessed with the CellChat package version 1.1.3. Gene set enrichment analysis and gene set variation analysis were performed with clusterProfiler version 4.6.2. Pseudotime trajectories were inferred with Monocle3 version 1.0.0. Marker gene expression was visualized with dot plots, violin plots, and heatmaps.

### Quantification and statistical analysis

Statistical analyses were conducted using Student's t-test for two-group comparisons (indicated by square brackets) and one-way ANOVA for multi-group experiments. Survival was evaluated by Kaplan-Meier analysis. Data are shown as mean ± SEM (n≥3). N.S.,not significant, p > 0.05, *p < 0.05, ** p < 0.01, ***p < 0.001, ****p < 0.0001.

### Data and software availability

All sequencing data have been deposited in Sequence Read Archive under accession number PRJNA1391677.

## Supplementary Material

Supplementary figures and tables.
