# Supplementary material for: Fangchinoline Activates cGAS-STING to Promote Antitumor Immunity without Pathological Inflammation
Source: Int J Biol Sci. 2026 May 18;22(10):5444–58. doi: 10.7150/ijbs.133255 (PMC13215355; doi:10.7150/ijbs.133255)
Supplement: Supplementary file 1 — Supplementary figures and tables. [file ijbsv22p5444s1.pdf]

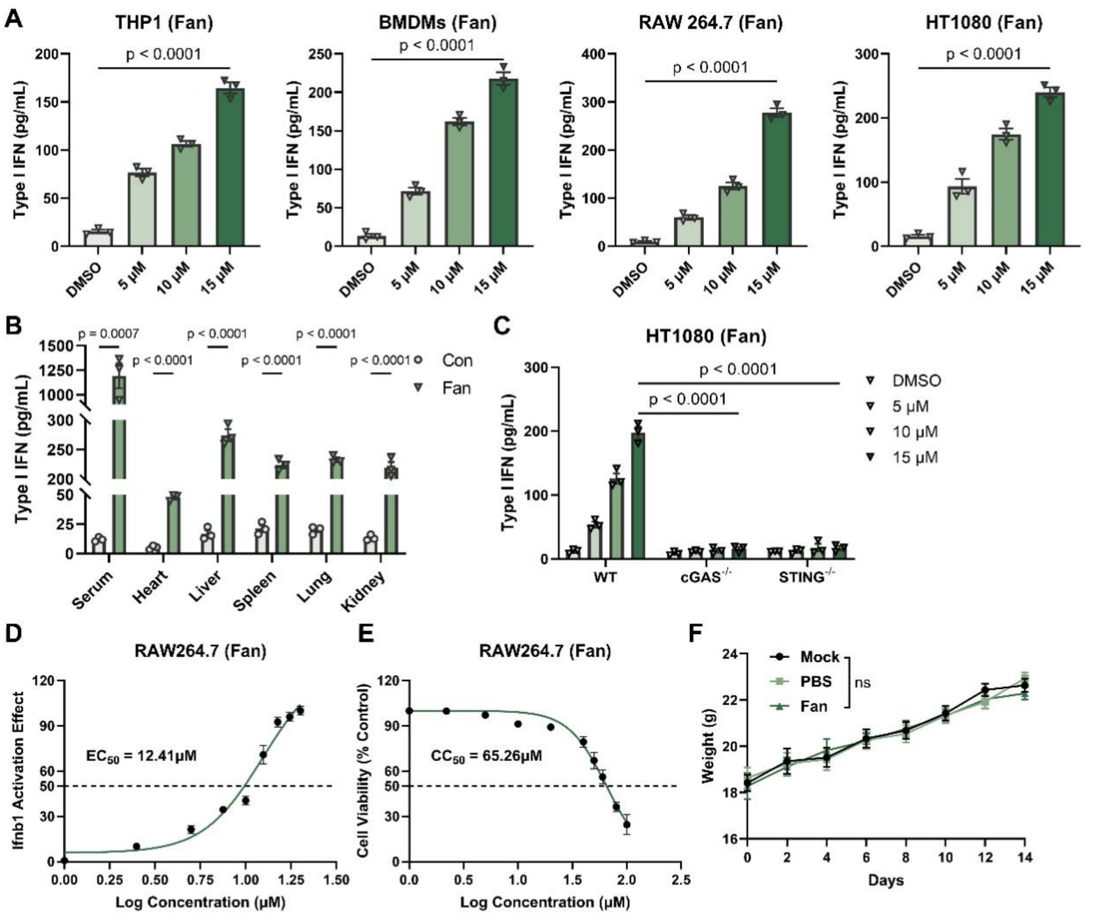

Supplementary Figure 1

(A) Secreted type I IFN measured by ELISA in the same THP1, BMDMs, RAW264.7 and HT1080 cells and treatments as in Figure 1A-1D. Ordinary one-way ANOVA test. (B) Secreted type I IFN measured by ELISA in mice serum and targeted organs. Six-week-old C57BL/6J mice 6 hours after a single intraperitoneal dose of Fan (20 mg/kg). Unpaired t test. (C) Secreted type I IFN measured by ELISA in the same cell lines and treatments as in Figure 1F. Two-way ANOVA test. (D) Dose-response analysis of Fan in RAW 264.7 cells. The apparent EC50 was determined based on *IFNB1* mRNA induction after treatment with the indicated concentrations of Fan. (E) The CC50 was determined in parallel using a cell viability assay under the same treatment conditions. (F) Body weight monitoring of C57BL/6J mice during two weeks of repeated intraperitoneal administration of Fan or vehicle control under the indicated dosing schedule. Two-way ANOVA test. Data are shown as mean ± SEM.

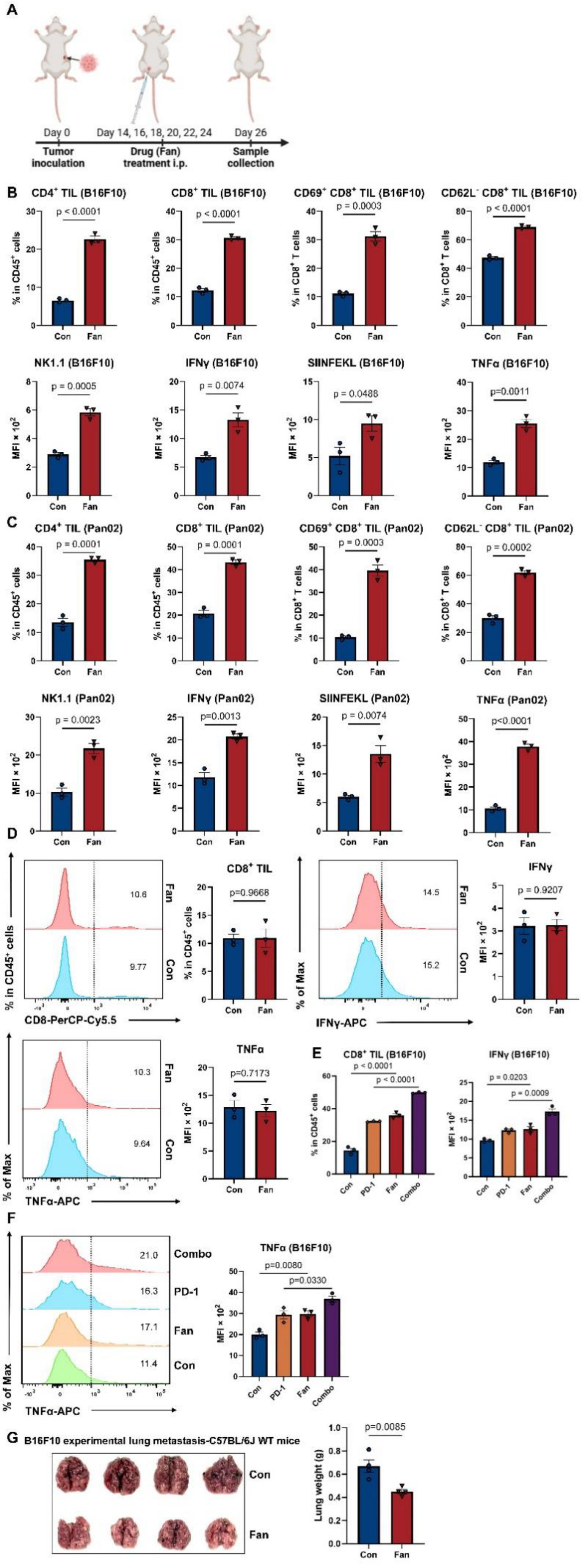
A
Day 0
Day 14, 16, 18, 20, 22, 24
Day 26
Tumor inoculation
Drug (Fan) treatment i.p.
Sample collection
B
CD4+ TIL (B16F10)
CD8+ TIL (B16F10)
CD69+ CD8+ TIL (B16F10)
CD62L- CD8+ TIL (B16F10)
NK1.1 (B16F10)
IFNγ (B16F10)
SIINFEKL (B16F10)
TNFα (B16F10)
C
CD4+ TIL (Pan02)
CD8+ TIL (Pan02)
CD69+ CD8+ TIL (Pan02)
CD62L- CD8+ TIL (Pan02)
NK1.1 (Pan02)
IFNγ (Pan02)
SIINFEKL (Pan02)
TNFα (Pan02)
D
CD8+ TIL
IFNγ
TNFα
CD8-PerCP-Cy5.5
IFNγ-APC
TNFα-APC
E
CD8+ TIL (B16F10)
IFNγ (B16F10)
F
TNFα (B16F10)
Combo
PD-1
Fan
Con
G
B16F10 experimental lung metastasis-C57BL/6J WT mice
Lung weight (g)

Supplementary Figure 2

(A) Schematic illustration of the experimental design for evaluating the therapeutic efficacy of Fan in ectopic tumor models. (B) Quantification of tumor infiltrating $CD45^{+}$ $CD4^{+}$ TILs, $CD45^{+}$ $CD8^{+}$ TILs, $CD69^{+}$ $CD8^{+}$ TILs, $CD62L^{-}$ $CD8^{+}$ TILs, $NK1.1^{+}$ TILs, $IFN\gamma^{+}$ TILs, $SIINFEKL^{+}$ TILs and $TNF\alpha^{+}$ TILs of mice as in Figure 2A. Unpaired t test. (C) Quantification of tumor infiltrating $CD45^{+}$ $CD4^{+}$ TILs, $CD45^{+}$ $CD8^{+}$ TILs, $CD69^{+}$ $CD8^{+}$ TILs, $CD62L^{-}$ $CD8^{+}$ TILs, $NK1.1^{+}$ TILs, $IFN\gamma^{+}$ TILs, $SIINFEKL^{+}$ TILs and $TNF\alpha^{+}$ TILs of mice as in Figure 2D. Unpaired t test. (D) Representative FACS data and quantification of tumor infiltrating $CD45^{+}$ $CD8^{+}$ TILs, $IFN\gamma^{+}$ TILs and $TNF\alpha^{+}$ TILs of mice as in Figure 2G. Unpaired t test. (E) Quantification of tumor infiltrating $CD45^{+}$ $CD8^{+}$ TILs, $IFN\gamma^{+}$ TILs of mice as in Figure 2I. Ordinary one-way ANOVA test. (F) Representative FACS data and quantification of tumor infiltrating $TNF\alpha^{+}$ TILs of mice as in Figure 2I. Ordinary one-way ANOVA test. (G) Representative images of lungs and lung weights at the experimental endpoint in the B16F10 lung metastasis model. Unpaired t test.Data are shown as mean ± SEM.

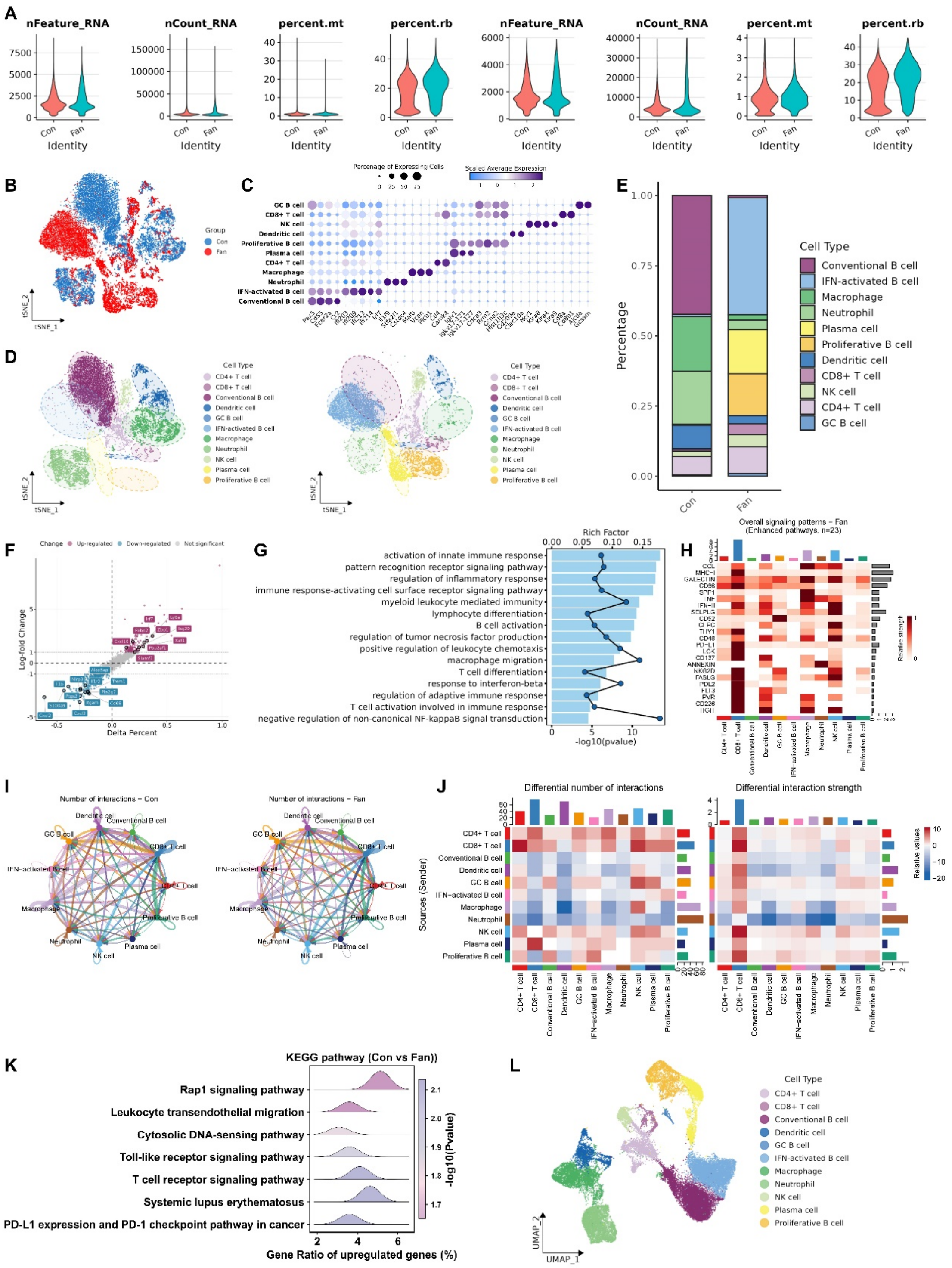

Supplementary Figure 3

(A) The panel showed pre-QC distributions of key quality metrics and the corresponding post-QC distributions following application of our filtering criteria. (B) t-SNE visualization of scRNA-seq data showing the distribution of immune cells from Con and Fan treatment groups. (C) Marker gene expression across the cell clusters was shown as a dot plot. The size and color of the dots respectively represent the proportion of cells expressing the corresponding genes and the average scaling value

of their logarithmic transformation expression. (D) t-SNE projection of scRNA-seq data annotated by major immune cell types. (E) Bar chart comparing the proportions of major cell types between Fan- and PBS-treated groups in $CD45^+$ B16F10 tumors. (F) Differential cell-type abundance analysis comparing Fan and Con groups. (G) Gene Ontology (GO) enrichment analysis of differentially expressed genes (DEGs) in $CD45^+$ cells following Fan treatment compared to Con. (H) Heatmap of gene expression across various cell types in the Fan- and PBS-treated groups. (I) Interaction network between different cell types in the tumor microenvironment, showing predicted interactions. Line thickness indicated the number of interactions or the strength of interactions. (J) Heatmap of differential number of interactions across various cell types in the Fan and Con groups. (K) KEGG pathway analysis of DEGs. (L) UMAP visualization of scRNA-seq data showing the distribution of immune cells from Con and Fan treatment groups.

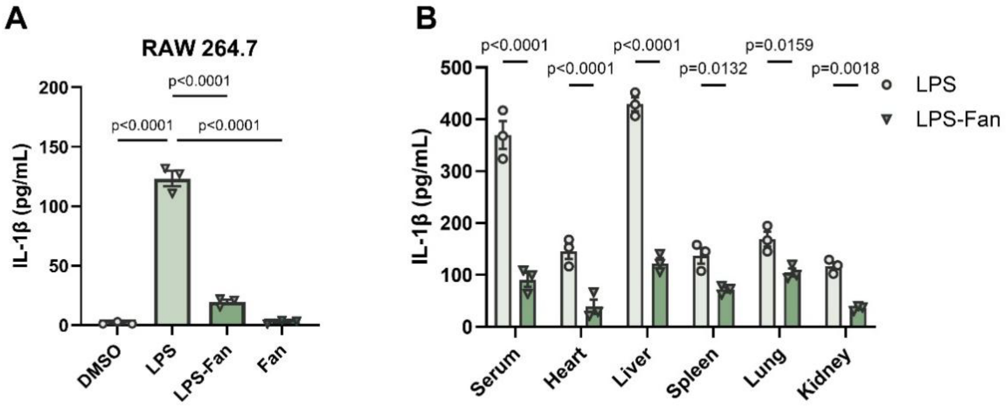

Supplementary Figure 4

(A) ELISA quantification of secreted IL-1β in the culture supernatants of RAW264.7 cells following treatment with DMSO, LPS, LPS+Fan, or Fan alone. Ordinary one-way ANOVA test. (B) ELISA quantification of IL-1β protein levels in serum, heart, liver, spleen, lung, and kidney from C57BL/6J mice following LPS challenge with or without Fan treatment. Unpaired t test. Data are shown as mean ± SEM.

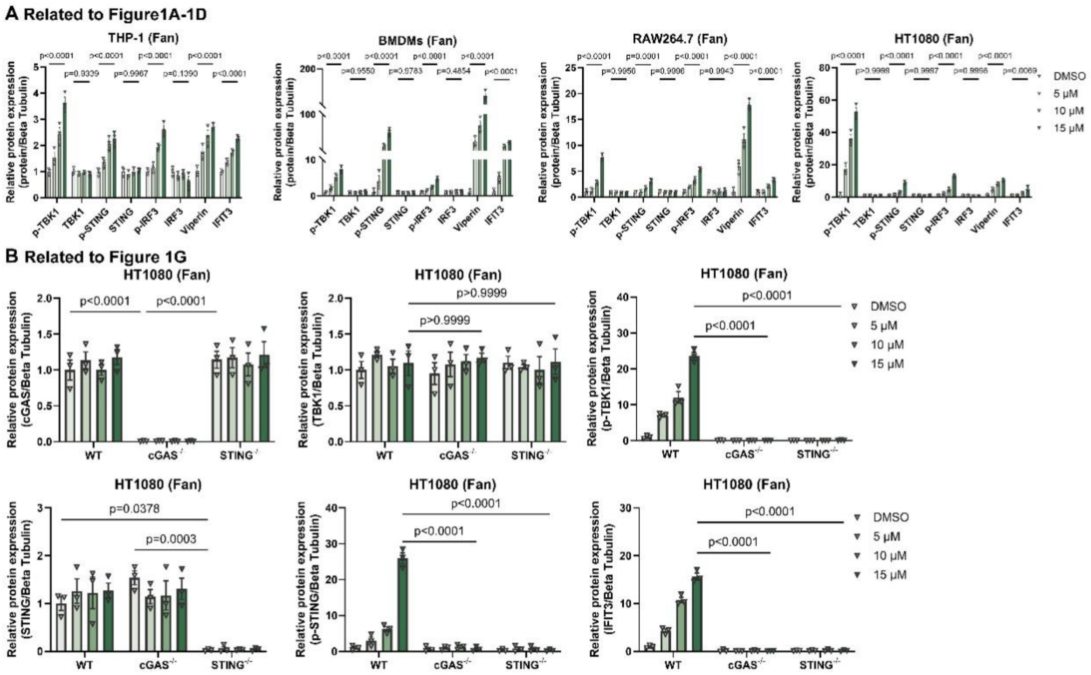

Supplementary Figure 5

(A-B) quantification of band intensities in Figure 1A-1D (A) and Figure 1G (B). Densitometry was performed in ImageJ 1.51 using fixed rectangular ROIs with local background subtraction. Target proteins were normalized to loading control and then to the vehicle group, which was set to 1.0. Data are shown as mean ± SEM (n = 3 independent experiments). Two-way ANOVA test. Data are shown as mean ± SEM.

Supplementary Table 1. Primer sequences used for RT-qPCR.

Forward and reverse primer sequences (5'→3') for all genes analyzed in this study.

Gene	Primer sequences
**hIFNB**	F: 5-GCTTGGATTCCTACAAAGAAGCA-3<br>R: 5-ATAGATGGTCAATGCGGCGTC-3
**mIl1b**	F: 5-AGTTGACGGACCCCAAA-3<br>R: 5-TCTTGTTGATGTGCTGCTG-3
**mIl6**	F: 5-CTGCAAGAGACTTCCATCCAG-3<br>R: 5-AGTGGTATAGACAGGTCTGTTGG-3
**mIfnb**	F: 5-TCCGAGCAGAGATCTTCAGGAA-3<br>R: 5-TGCAACCACCACTCATTCTGAG-3
**hACTB**	F: 5-CCTGGCACCCAGCACAAT-3<br>R: 5-GCCGATCCACACGGAGTACT-3
**mActb**	F: 5-GGCTGTATTCCCCTCCATCG-3<br>R: 5-CCAGTTGGTAACAATGCCATGT-3
**mNos2**	F: 5-GTTCTCAGCCCAACAATACAAGA-3<br>R: 5-GTGGACGGGTCGATGTCAC-3
**mPtgs2**	F: 5-TTCAACACACTCTATCACTGGC-3<br>R: 5-AGAAGCGTTTGCGGTACTCAT-3
**mLta**	F: 5-CCACCTCTTGAGGGTGCTTG-3<br>R: 5-CATGTCGGAGAAAGGCACGAT-3

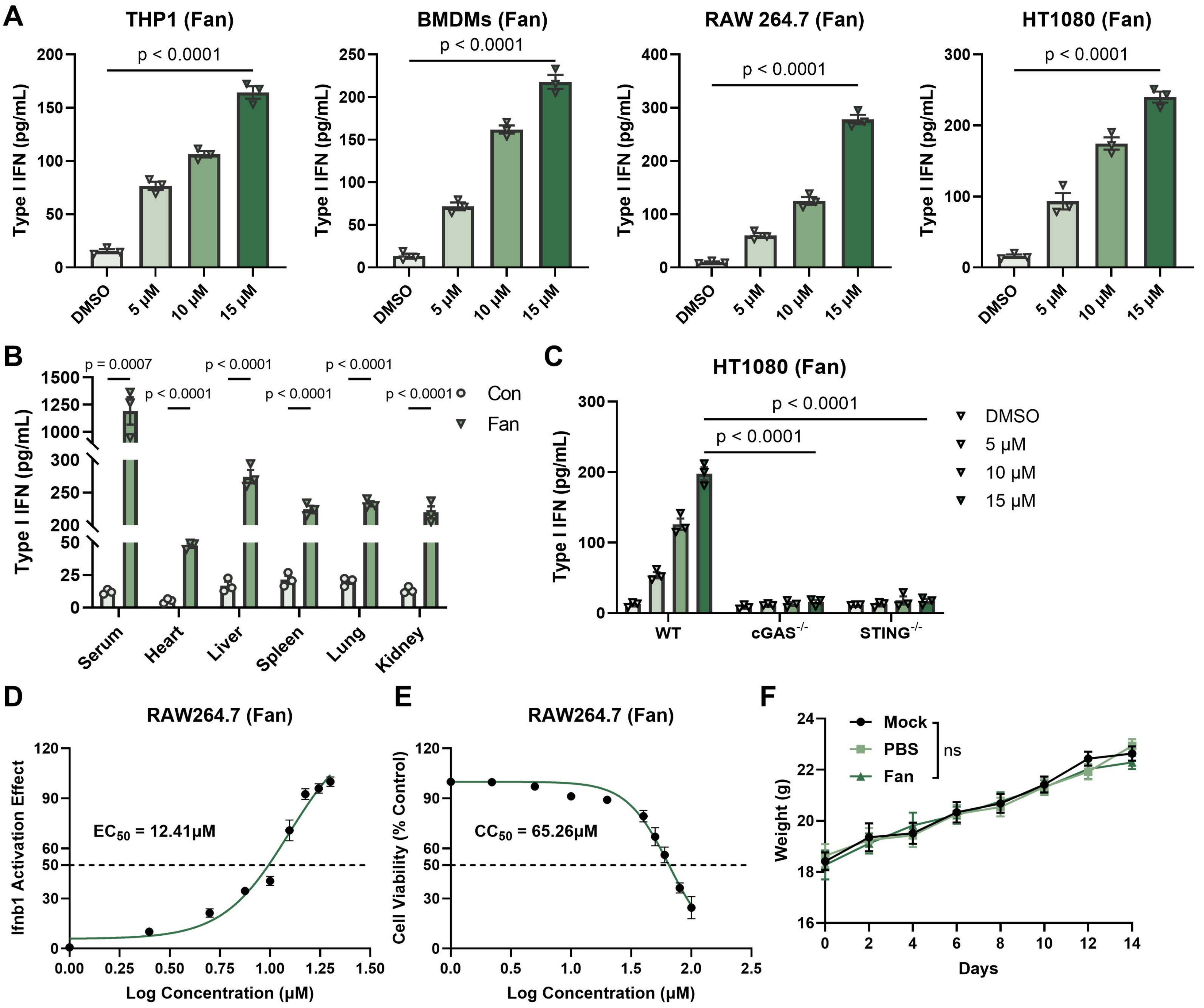

A
THP1 (Fan)
BMDMs (Fan)
RAW 264.7 (Fan)
HT1080 (Fan)
p < 0.0001
Type I IFN (pg/mL)
DMSO
5 μM
10 μM
15 μM
B
p = 0.0007
p < 0.0001
Con
Fan
Serum
Heart
Liver
Spleen
Lung
Kidney
C
HT1080 (Fan)
DMSO
5 μM
10 μM
15 μM
WT
cGAS-/-
STING-/-
D
RAW264.7 (Fan)
Ifnb1 Activation Effect
EC50 = 12.41μM
Log Concentration (μM)
E
RAW264.7 (Fan)
Cell Viability (% Control)
CC50 = 65.26μM
Log Concentration (μM)
F
Mock
PBS
Fan
ns
Weight (g)
Days

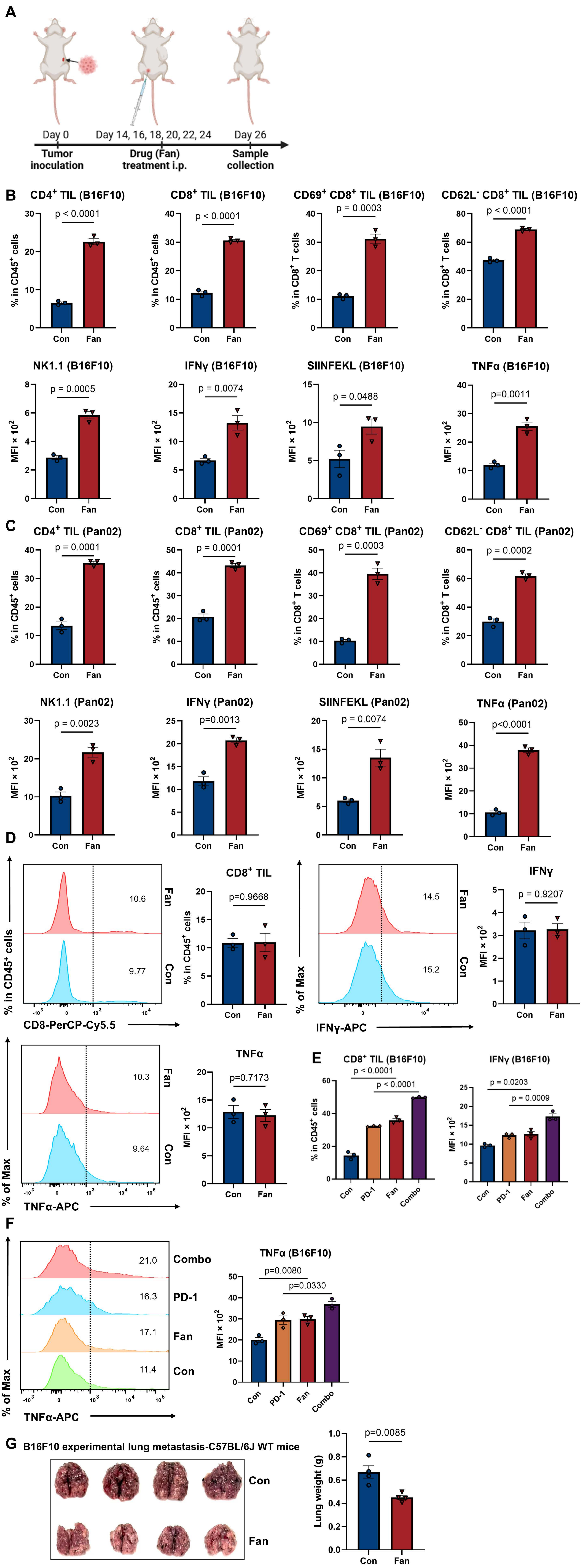
A
Day 0
Tumor inoculation
Day 14, 16, 18, 20, 22, 24
Drug (Fan) treatment i.p.
Day 26
Sample collection
B
CD4+ TIL (B16F10)
p < 0.0001
CD8+ TIL (B16F10)
p < 0.0001
CD69+ CD8+ TIL (B16F10)
p = 0.0003
CD62L- CD8+ TIL (B16F10)
p < 0.0001
% in CD45+ cells
% in CD8+ T cells
NK1.1 (B16F10)
p = 0.0005
IFNγ (B16F10)
p = 0.0074
SIINFEKL (B16F10)
p = 0.0488
TNFα (B16F10)
p=0.0011
MFI × 10^2
Con
Fan
C
CD4+ TIL (Pan02)
p = 0.0001
CD8+ TIL (Pan02)
p = 0.0001
CD69+ CD8+ TIL (Pan02)
p = 0.0003
CD62L- CD8+ TIL (Pan02)
p = 0.0002
NK1.1 (Pan02)
p = 0.0023
IFNγ (Pan02)
p=0.0013
SIINFEKL (Pan02)
p = 0.0074
TNFα (Pan02)
p<0.0001
D
% in CD45+ cells
10.6
9.77
CD8-PerCP-Cy5.5
CD8+ TIL
p=0.9668
% of Max
14.5
15.2
IFNγ-APC
IFNγ
p = 0.9207
10.3
9.64
TNFα-APC
TNFα
p=0.7173
E
CD8+ TIL (B16F10)
p < 0.0001
p < 0.0001
IFNγ (B16F10)
p = 0.0203
p = 0.0009
Con
PD-1
Fan
Combo
F
21.0
16.3
17.1
11.4
TNFα (B16F10)
p=0.0080
p=0.0330
G
B16F10 experimental lung metastasis-C57BL/6J WT mice
Con
Fan
p=0.0085
Lung weight (g)

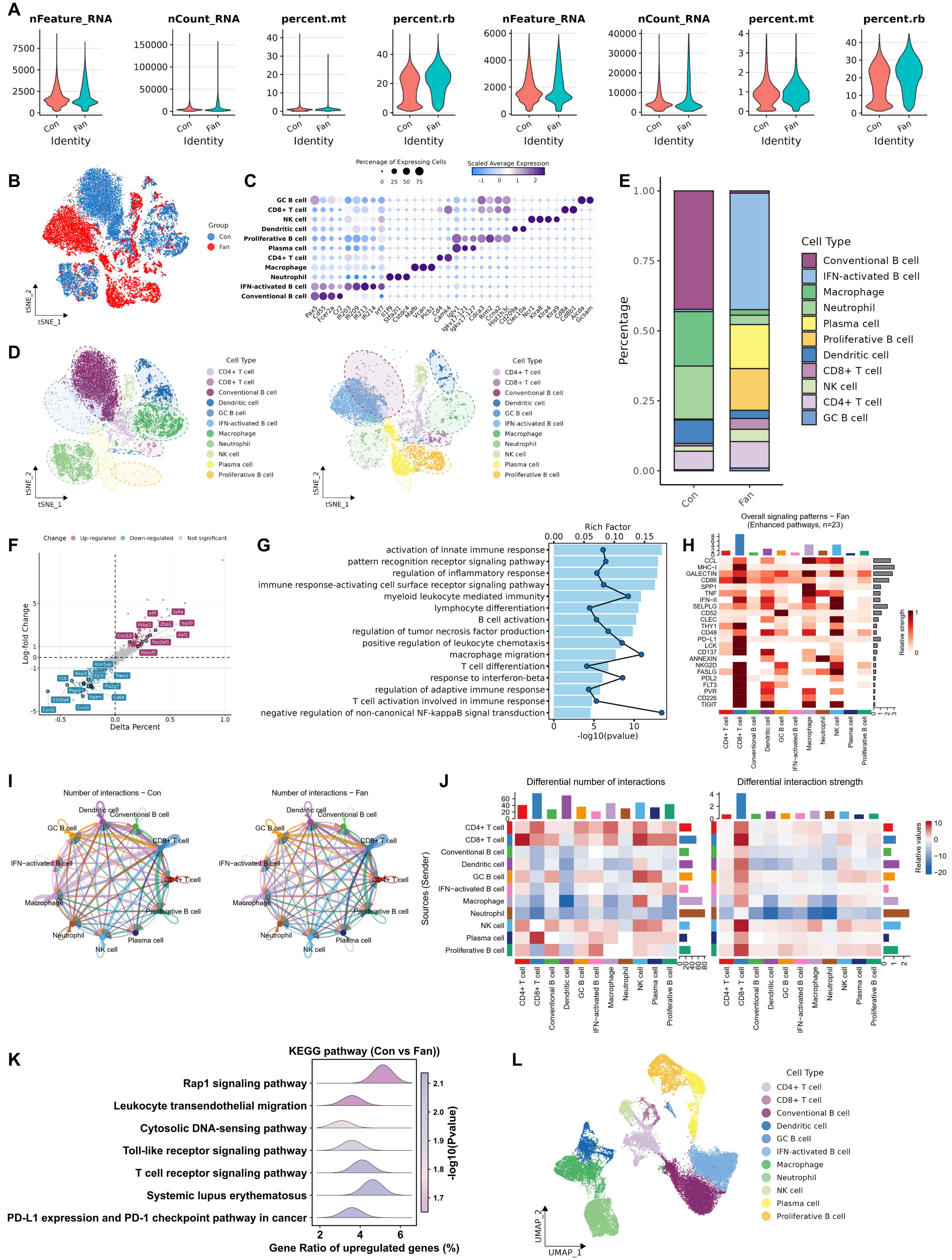
A
nFeature_RNA
nCount_RNA
percent.mt
percent.rb
Con
Fan
Identity
B
Group
Con
Fan
tSNE_1
tSNE_2
C
Percenage of Expressing Cells
Scaled Average Expression
GC B cell
CD8+ T cell
NK cell
Dendritic cell
Proliferative B cell
Plasma cell
CD4+ T cell
Macrophage
Neutrophil
IFN-activated B cell
Conventional B cell
D
Cell Type
E
Percentage
Cell Type
Conventional B cell
IFN-activated B cell
Macrophage
Neutrophil
Plasma cell
Proliferative B cell
Dendritic cell
CD8+ T cell
NK cell
CD4+ T cell
GC B cell
F
Change
Up-regulated
Down-regulated
Not significant
Log-fold Change
Delta Percent
G
Rich Factor
activation of innate immune response
pattern recognition receptor signaling pathway
regulation of inflammatory response
immune response-activating cell surface receptor signaling pathway
myeloid leukocyte mediated immunity
lymphocyte differentiation
B cell activation
regulation of tumor necrosis factor production
positive regulation of leukocyte chemotaxis
macrophage migration
T cell differentiation
response to interferon-beta
regulation of adaptive immune response
T cell activation involved in immune response
negative regulation of non-canonical NF-kappaB signal transduction
-log10(pvalue)
H
Overall signaling patterns − Fan
(Enhanced pathways, n=23)
Relative strength
I
Number of interactions − Con
Number of interactions − Fan
J
Differential number of interactions
Differential interaction strength
Sources (Sender)
Relative values
K
KEGG pathway (Con vs Fan))
Rap1 signaling pathway
Leukocyte transendothelial migration
Cytosolic DNA-sensing pathway
Toll-like receptor signaling pathway
T cell receptor signaling pathway
Systemic lupus erythematosus
PD-L1 expression and PD-1 checkpoint pathway in cancer
-log10(Pvalue)
Gene Ratio of upregulated genes (%)
L
Cell Type
UMAP_1
UMAP_2

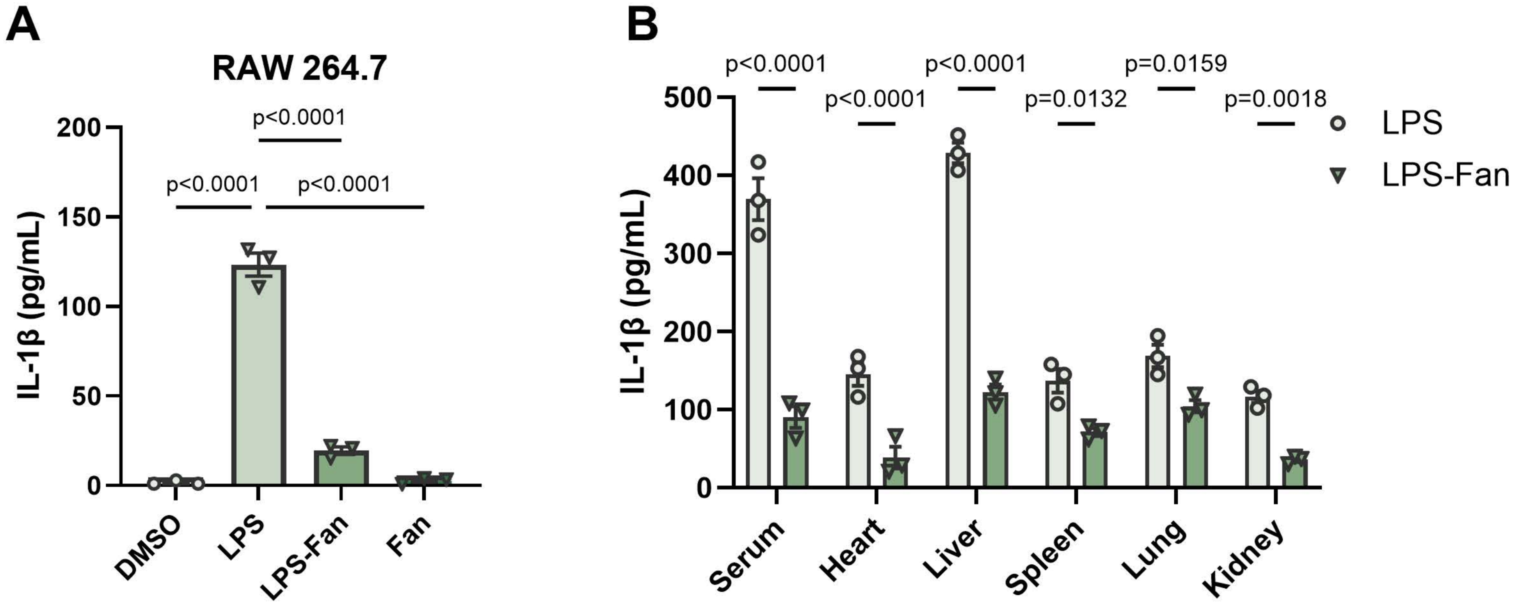
A
RAW 264.7
IL-1β (pg/mL)
200
150
100
50
0
p<0.0001
p<0.0001
p<0.0001
DMSO
LPS
LPS-Fan
Fan
B
IL-1β (pg/mL)
500
400
300
200
100
0
p<0.0001
p<0.0001
p<0.0001
p=0.0132
p=0.0159
p=0.0018
LPS
LPS-Fan
Serum
Heart
Liver
Spleen
Lung
Kidney

A Related to Figure1A-1D

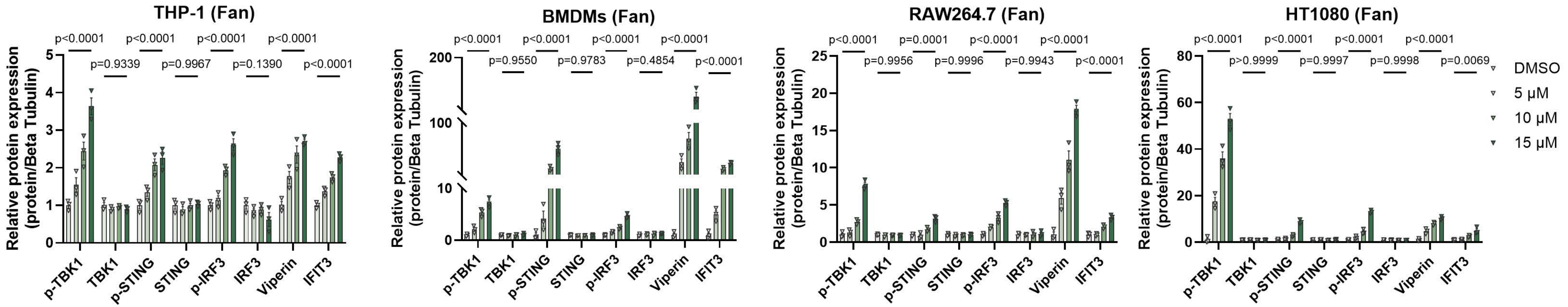

B Related to Figure 1G

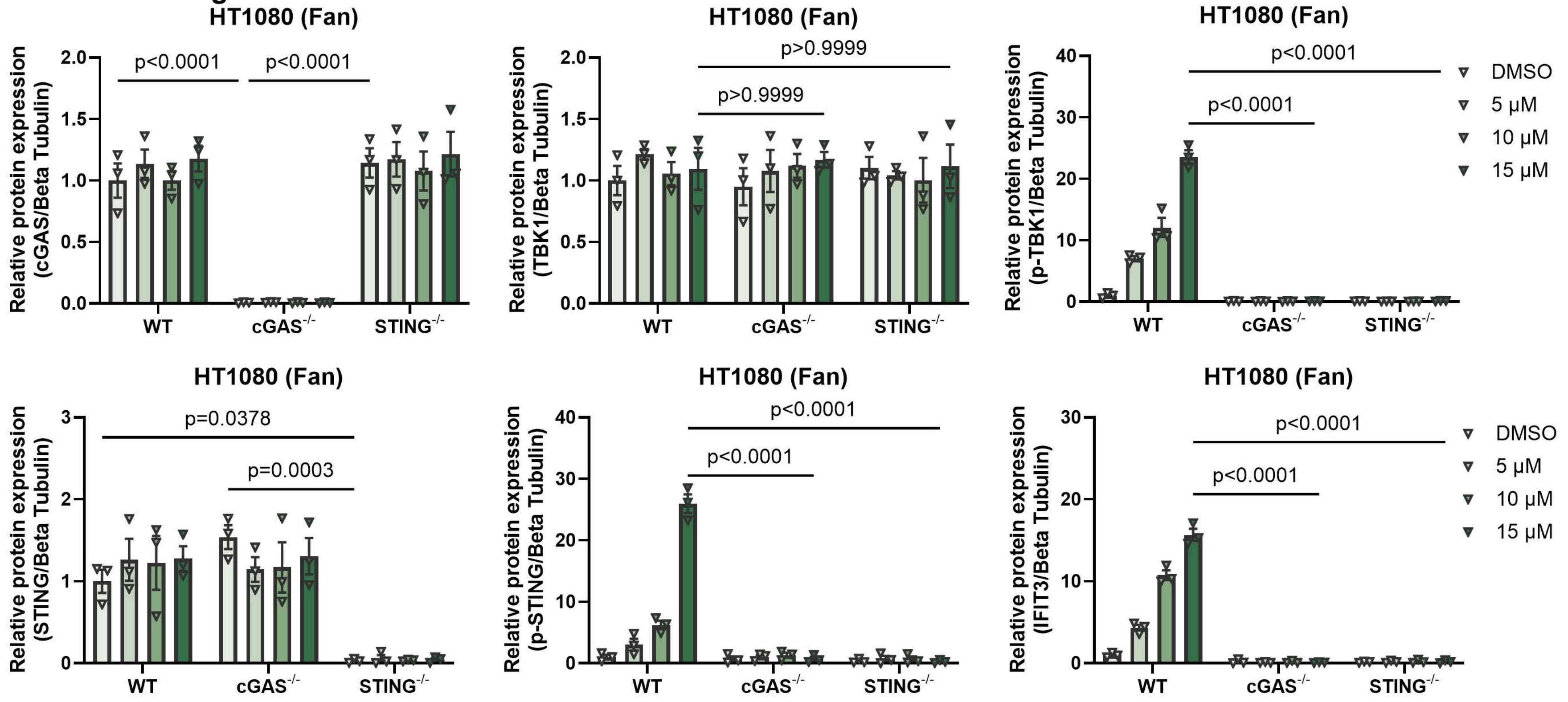

Supplementary Table 1. Primer sequences used for RT-qPCR.

gene	primer sequences
hIFNB-F	GCTTGGATTCCTACAAAGAAGCA
hIFNB-R	ATAGATGGTCAATGCGGCGTC
mIl1b-F	AGTTGACGGACCCCAAA
mIl1b-R	TCTTGTTGATGTGCTGCTG
mIl6-F	CTGCAAGAGACTTCCATCCAG
mIl6-R	AGTGGTATAGACAGGTCTGTTGG
mIfnb-F	TCCGAGCAGAGATCTTCAGGAA
mIfnb-R	TGCAACCACCACTCATTCTGAG
hACTB-F	CCTGGCACCCAGCACAAT
hACTB-R	GCCGATCCACACGGAGTACT
mActb-F	GGCTGTATTCCCCTCCATCG
mActb-R	CCAGTTGGTAACAATGCCATGT
mNos2_F	GTTCTCAGCCCAACAATACAAGA
mNos2_F	GTGGACGGGTCGATGTCAC
mPtgs2_[illegible]	TTCAACACACTCTATCACTGGC
mPtgs2_[illegible]	AGAAGCGTTTGCGGTACTCAT
mLta_F	CCACCTCTTGAGGGTGCTTG
mLta_R	CATGTCGGAGAAAGGCACGAT
